# Cancer Cell Metabolic Reprogramming: Dietary, Pharmacological Strategies and Clinical Perspectives

**DOI:** 10.3390/cancers18203372

**Published:** 2026-10-18

**Authors:** Nikolaos Xydas, Emmanouil Panagiotou, Vasileios Kassos, Athina Maria Gkirmpa, Paraskevi Kassou, Ioannis Voyiatzis, Paoula Prenti, George Manias, Aigli Vakrakou, Athanasios K. Anagnostopoulos

**Affiliations:** 1 Global Business School for Health, University College London, London WC1E 6BT, UK; 2 Third Department of Internal Medicine, Sotiria General Hospital for Chest Diseases, National and Kapodistrian University of Athens, 11527 Athens, Greece; 3 Department of Basic and Clinical Sciences, Medical School, University of Nicosia, UNIC Athens, 16777 Athens, Greece; kassos.v@live.unic.ac.cy; 4 Department of Pathology and Surgery, Hygeia Hospital, 15123 Athens, Greece; 5 Division of Biotechnology, Biomedical Research Foundation of the Academy of Athens, 11527 Athens, Greece; 6 Department of Digital Systems, University of Piraeus, 18534 Piraeus, Greece; 7 First Department of Neurology, Aeginition Hospital, National and Kapodistrian University of Athens, 11528 Athens, Greece

**Keywords:** metabolic reprogramming, cancer, proteomics, metabolomics, nutrition, IGF-1, mTOR, tumor microenvironment, mass spectrometry, LC-MS/MS

## Abstract

Metabolic reprogramming, a key feature of cancer progression that enables tumor adaptation to stress and therapy, represents an emerging target in anticancer research through dietary and pharmacological modulation. Dietary strategies such as caloric restriction (CR), intermittent fasting, and nutrient-specific modifications are emerging approaches to target oncogenic metabolism. Preclinical studies show that CR reduces tumor burden and inhibits IGF-1/mTOR signaling, while restoring immune pathways. Complementary preclinical evidence confirms that pharmacological inhibitors of the IGF-1R and mTOR pathways independently expose metabolic vulnerabilities, while concurrent autophagy blockade alongside mTOR inhibition can further reduce cancer cell viability by preventing stress adaptation. Multi-omics analyses facilitate the identification of how dietary restriction and pharmacological interventions remodel the tumor microenvironment by altering immune infiltration and stromal interactions, and uncover metabolic dependencies, that represent actionable therapeutic vulnerabilities. These findings suggest that dietary and pharmacological metabolic modulation represents an innovative approach in anticancer research. Preclinical models demonstrate the capability of these interventions to suppress tumor growth and enhance therapeutic responses, while early-phase clinical trials currently establish their safety and biological feasibility. In this review, we aim to summarize findings from in vitro models, in vivo animal studies, clinical observations, and multi-omics analyses to assess the potential of diet and pharmacological mimetics in influencing tumor metabolism and the tumor microenvironment.

## Simple Summary

Cancer cells change the way they use nutrients and produce energy in order to grow, survive, and resist treatment. Targeting these metabolic changes through dietary changes or drugs is therefore emerging as a promising approach in cancer research. In this review, we explain how changing the calories or the nutrients of the food we consume, eating only at certain times of the day, or drugs that have similar effects as those dietary changes may influence tumor growth and the tissues around the tumor. We also discuss how modern technologies can help identify changes caused by these interventions on the molecular and metabolic level. Evidence from laboratory studies, animal models, and clinical observations suggests that modifying cancer metabolism may help slow tumor progression and improve responses to existing treatments. Further clinical research is needed to determine how these strategies can be safely and effectively incorporated into cancer care.

## 1. Introduction

Dietary intake is a major determinant of metabolic function and overall health. Nutrient availability influences not only cellular energy production but also gene expression, signaling activity, and long-term physiological adaptation [1]. Metabolic reprogramming refers to the ability of cells to alter their metabolic behavior in response to changing biological demands or environmental conditions [2]. Rather than being a single metabolic change, it is a coordinated process through which cells adjust how they use available nutrients to support growth, survival, repair, or adaptation to stress [3]. In disease, metabolic regulation serves as a central control point linking nutrient utilization to cellular behavior. When this regulation is disrupted, cells can preserve abnormal functions that contribute to disease progression [4]. Cancer is characterized by the continuous metabolic adaptation of malignant cells to support uncontrolled growth under fluctuating nutrient and oxygen conditions [5].

Metabolic reprogramming is recognized as a core hallmark of cancer, facilitating malignant cell survival and sustained proliferation through alterations in metabolic enzyme activity, upstream regulatory pathways, and downstream metabolites [6,7]. This process is shaped by multiple factors, including oncogene activation, tumor suppressor loss, growth-factor signaling, and tumor–host cell interactions, which together support the anabolic requirements of cancer cells and promote tumor growth [8]. These interactions occur within the tumor microenvironment (TME), the local biological niche in which tumor cells interact with neighboring cells, tissue structures, and soluble signals. The TME actively shapes tumor behavior by influencing nutrient availability, oxygen supply, cellular signaling, and metabolic stress [9]. In this environment, malignant cells reorganize nutrient acquisition in response to hypoxia and nutrient deprivation, allowing them to meet their bioenergetic and redox demands under metabolically challenging conditions [8].

Malignant cells alter their metabolism to meet the demands of unregulated proliferation, survival, and migration. These processes require a continuous supply of energy, biosynthetic precursors, and signaling intermediates [10]. Accordingly, cancer cell metabolic reprogramming is driven by both intrinsic factors, including oncogenic mutations, transcriptional changes, and epigenetic modifications, and extrinsic environmental pressures that shape the conditions in which tumor cells grow [11,12]. Tumor cell metabolic alterations are not limited to upregulation of isolated pathways; instead, they integrate metabolic activity with other cellular functions through spatial and temporal modulation of metabolic enzymes [13]. Distinct tumor cell populations may rely on different metabolic programs, as illustrated by invasive H1299 (non-small cell lung cancer) cell clusters in which the subpopulations of highly invasive cells preferentially engage mitochondrial respiration in vitro, whereas poorly invasive cells show increased glucose uptake and glucose transporter 1 (GLUT1) expression [14]. These metabolic states may evolve during tumor progression and in response to environmental pressures, contributing to cancer aggressiveness, adaptation, and treatment resistance [15].

Nutrient availability is one of the main factors shaping cellular metabolism; therefore, diet and nutrition provide a direct connection between the host metabolic state and tumor behavior, as dietary intake can influence both the metabolic environment in which cancer cells grow and the signaling networks that regulate their behavior [16]. Nutrient availability within the TME strongly influences cancer metabolism, and dietary modifications targeting oncogenic metabolic pathways may partially modulate this metabolic state [17]. Since tumors depend on host-derived nutrients for growth and survival, dietary interventions may alter nutrient availability within the TME and thereby influence tumor behavior. By changing the metabolic conditions on which tumor cells depend, such interventions may also enhance anticancer drug efficacy by exposing metabolic vulnerabilities and modifying nutrient-sensitive signaling pathways [18]. In this context, nutrient sensing is central to tumor evolution, allowing cancer cells to adjust their metabolic output in response to changing nutrient conditions through nutrient-responsive transcription factors and signaling pathways [19]. Collectively, these observations provide the rationale for targeting cancer metabolic reprogramming through both dietary and pharmacological strategies. However, because tumor metabolism is heterogeneous, spatially organised, and influenced by both tumor-intrinsic and host-derived factors, its therapeutic modulation can take advantage of integrated multi-omics approaches [20].

This review aims to critically evaluate cancer cell metabolic reprogramming as a diet- and nutrition-sensitive therapeutic vulnerability, focusing on how nutrient availability, pharmacological mimetics, and multi-omics profiling can be integrated to guide more precise metabolic approaches in oncology.

## 2. Materials and Methods

We conducted a literature search of PubMed, Google Scholar, Web of Science, and ClinicalTrials.gov from inception to July 2026. The search strategy employed combinations of core terms including “cancer”, “tumor”, “metabolic reprogramming” and “cancer metabolism”. To explore nutritional interventions, these were combined with terms such as “diet”, “nutrition”, “caloric restriction”, “fasting”, “fasting-mimicking diet” and “nutrient modulation”. Additional domain-specific keywords, including “autophagy”, “tumor microenvironment”, “metabolomics”, “single-cell,” and “spatial omics” were utilized to capture the relevant mechanistic and multi-omics literature. The reference lists of eligible articles and relevant clinical trial registries were additionally screened to identify pertinent studies.

Two independent reviewers (N.X. and E.P.) screened the titles and abstracts, identifying articles suitable for full-text review. Disagreements regarding inclusion were resolved through consensus mediation by the senior author (A.A.). Articles were evaluated and selected based on their conceptual relevance to the narrative review topic. Included studies were required to be full-text articles published in English that investigated cancer metabolic reprogramming in the context of dietary, nutritional, or nutrient-targeted pharmacological interventions. Priority was given to studies providing robust mechanistic, in vitro, in vivo, clinical, or advanced omics (e.g., single-cell, spatial) evidence. We focused on extracting thematic findings related to specific metabolic variables (e.g., glucose, insulin, insulin-like growth factor 1 [IGF-1], lactate, reactive oxygen species), core signaling pathways (e.g., PI3K-AKT-mTOR, hypoxia-inducible factor 1 alpha [HIF-1α]), and primary outcomes such as tumor growth, treatment sensitivity, and tumor microenvironment remodeling. Studies were excluded if they lacked direct relevance to cancer metabolism, provided insufficient mechanistic information, or presented duplicate data. The selected literature was qualitatively synthesized to provide a comprehensive, thematic overview of the field. Given the diverse experimental approaches evaluated in this review, Table 1 summarizes common in vivo animal models and Table 2 outlines representative in vitro cellular models that form the basis of the compiled mechanistic evidence.

## 3. Metabolic Reprogramming and the Tumor Microenvironment

Rather than functioning as a passive background, the TME actively shapes tumor progression by influencing nutrient availability, oxygen delivery, inflammatory signaling, immune surveillance, and treatment response [33]. This is particularly relevant to cancer metabolic reprogramming, as metabolic changes in tumor cells are shaped by local environmental pressures and, in turn, remodel the conditions that support tumor growth, immune escape, and therapeutic resistance [34].

One major feature of TME remodeling is the formation of spatially distinct immune niches. Immune-infiltrated regions can support interactions among tumor cells, cytotoxic T cells, and antigen-presenting cells, whereas immune-excluded regions are characterized by impaired entry of lymphocytes into tumor nests [35]. The presence of immune cells in the surrounding stroma does not necessarily indicate effective antitumor immunity; for immune-mediated tumor control, cytotoxic lymphocytes must be able to enter tumor regions, remain functionally active, and overcome local metabolic and stromal barriers [36,37].

Stromal crosstalk is, therefore, a central mechanism through which metabolic reprogramming reshapes the TME. Cancer-associated fibroblasts (CAFs) can influence tumor behavior through extracellular matrix remodeling, soluble signaling, and metabolic exchange with cancer cells [38]. Recent primary evidence highlights lactate as an important mediator of this stromal metabolic interaction. It is known that tumor cells preferentially metabolize glucose through glycolysis rather than oxidative phosphorylation, through a phenomenon described as the Warburg effect; this process creates large concentrations of intracellular lactate, which in turn is secreted and contributes to the acidification of the TME [39]. Affinito et al. showed that breast cancer CAFs express higher levels of monocarboxylate transporter 4 (MCT4) than normal fibroblasts and secrete greater amounts of lactate into the extracellular environment; disrupting CAF lactate export can weaken the pro-tumorigenic metabolic support provided by the stroma to tumor epithelial cells [31]. Similar evidence has been reported in colorectal cancer, where tumor cells and CAFs can establish lactate-mediated metabolic coupling [21]. Wang et al. showed that colorectal cancer cells induced metabolic reprogramming in fibroblasts through oxidative stress, promoting lactate production by CAF-like fibroblasts and lactate uptake by tumor cells through monocarboxylate transporter-dependent exchange; functionally, this lactate shuttle enhanced colorectal cancer cell invasion, migration, and angiogenic potential [21]. This supports the broader idea that lactate is not only a waste product of glycolysis but also a signaling and metabolic mediator linking tumor-cell metabolism to stromal remodeling. Tumor-infiltrating effector T cells preferentially utilize aerobic glycolysis, leading to competition with tumor cells for glucose or other nutrients within the TME. This nutrient restriction can impair T cell metabolism and suppress anti-tumor effector function [40]. In addition, glycolysis-associated changes in the TME can further inhibit T cell activity and promote immune evasion; targeting lactate production or secretion may provide a novel strategy to enhance cancer immunotherapy, although clinical evidence is currently limited [41].

Hypoxia is another major driver of TME remodeling. As tumors expand, abnormal vascular organization and insufficient oxygen delivery generate hypoxic regions that force cancer cells to reorganize their metabolism. Under these conditions, cancer cells frequently increase glycolytic activity, accumulate acidic metabolites, and activate hypoxia-responsive signaling pathways [42]. HIF-1α is particularly important because it links low oxygen availability to metabolic adaptation, angiogenesis, immune evasion, and treatment resistance [43]. The link between hypoxia and immune suppression is supported by evidence that HIF-1α can regulate programmed death-ligand 1 (PD-L1) expression in both tumor and myeloid compartments. In colorectal cancer, Sun et al. showed that HIF-1α and PD-L1 were increased in colorectal tumor tissue compared with adjacent normal tissue and were positively correlated; in vitro, induced hypoxia-like conditions increased HIF-1α and PD-L1 expression, while the HIF-1α inhibitor YC-1 reduced PD-L1 expression under these conditions [32]. Bailey et al. provided stronger in vivo evidence [26]. HIF-1α inhibition reduced PD-L1 expression in tumor cells and tumor-infiltrating myeloid populations; targeting the HIF-1α/PD-L1 axis reactivated tumor-infiltrating lymphocytes, reduced T cell exhaustion or apoptosis markers, and contributed to tumor rejection [26]. Hypoxia-inducible factor 2 alpha (HIF-2α), in contrast, has been linked to hypoxia-induced upregulation of cell proliferation in highly vascularized or hypoxic tissues, particularly after loss of function of the von Hippel–Lindau (VHL) protein [44]. The HIF-2α inhibitor belzutifan was initially approved in tumors associated with VHL disease after the LITESPARK-004 study [45], and was subsequently approved for clear cell renal cell carcinoma, which is commonly associated with alterations in the *VHL* gene, either as monotherapy or in combination with immunotherapy [46,47]. These findings support the idea that hypoxia is not only a metabolic stress condition but also an immunoregulatory force within the TME. They also provide a mechanistic bridge between metabolic stress, immune checkpoint regulation, and therapeutic response within the TME.

Metabolic stress can also reshape the physical structure of the TME. Acidic conditions, which often arise from increased glycolysis and lactate accumulation, can influence extracellular matrix organization and create physical barriers that limit immune-cell access to tumor nests [39]. Bai et al. provided direct primary evidence for this mechanism in colon cancer by showing that an acidic microenvironment activates GPR4, a proton-sensing receptor, driving collagen remodeling and promoting immune exclusion [27]. High GPR4 expression was associated with reduced intratumoral CD8-positive T cell infiltration despite stromal immune presence, consistent with an immune-excluded phenotype [27]. Tumor-associated extracellular matrix conditioning also promotes metabolic changes in tumor-associated macrophages, contributing to a metabolic environment that is unfavorable to T cell activity; these metabolic changes, mediated by transforming growth factor-β, also promote collagen deposition and TME fibrosis, further contributing to immune exclusion [48].

Overall, the TME should be understood as a metabolically active ecosystem in which tumor cells, immune cells, CAFs, vascular structures, and extracellular matrix components continuously influence one another. Metabolic reprogramming within this ecosystem affects not only tumor cell survival but also immune infiltration, stromal organization, hypoxia-associated signaling, and therapeutic resistance. These findings support the rationale for combining dietary or pharmacological metabolic strategies with approaches that target the TME. However, the evidence remains context-specific and requires further validation using in vivo models, spatial-omics, and clinical data, to link metabolic intervention to immune infiltration, stromal remodeling, hypoxia reversal, and treatment response.

## 4. Multi-Omics Insights into Metabolic Reprogramming

Multi-omics technologies provide a systems-level framework for integrating genomic, transcriptomic, proteomic, metabolomic, lipidomic, single-cell, and spatial data. This is particularly important in cancer metabolic reprogramming because metabolic pathway activity is not uniform across the tumor mass; instead, it varies according to tumor region, cell type, immune composition, stromal organization, nutrient availability, and therapeutic pressure [20]. As a result, bulk or single-marker approaches are often insufficient to capture the spatially heterogeneous, temporally dynamic, and cell-type-specific nature of cancer metabolism [49].

Single-cell sequencing has become central to this framework because it enables analysis of molecular features at the level of individual cells rather than averaging across a mixed cell population. Single-cell RNA sequencing (scRNA-seq) can resolve differences in gene expression across tumor, immune, stromal, and endothelial compartments, making it particularly useful for studying tumor heterogeneity, clonal evolution, immune states, and cell–cell communication within the tumor microenvironment. In the context of metabolic reprogramming, this approach can identify which cell populations express nutrient transporters, metabolic enzymes, stress-response pathways, or immune-regulatory programs, thereby linking metabolic state to cellular identity and function [50,51].

Rare immune or stromal populations may be missed by bulk profiling, but they can still shape local immune suppression, metabolic competition, or therapeutic response; recent studies have demonstrated the value of single-cell approaches for resolving cellular heterogeneity within the TME. Zhang et al. used single-cell technologies to characterize stromal and immune diversity, identify rare cellular subsets such as dendritic cell populations, and map signaling interactions associated with tumor progression [52]. Single-cell proteomic approaches can provide complementary information by measuring protein-level phenotypes that are not always predicted by transcriptomic data. Feng et al. used mass cytometry (CyTOF) to profile muscle-invasive bladder cancer at single-cell resolution, revealing phenotypic diversity across tumor epithelial and immune compartments and identifying tumor states and immune phenotypes that may inform biomarker discovery [53]. Through CyTOF-based clustering, the authors identified distinct epithelial tumor-cell populations with differential expression of markers associated with metastasis, immune checkpoints, stemness, and cancer-related signaling pathways. Nevertheless, claims linking these cell states directly to metabolic dependency, treatment resistance, or disease progression require additional experimental validation, such as cell depletion, co-culture assays, organoid models, or in vivo perturbation studies [53,54].

Spatial omics extends this analysis by preserving the anatomical context of tumor tissue. Spatial metabolomics, in particular, enables in situ detection and mapping of metabolites across different regions of a tissue section. This is highly relevant to cancer metabolism, as nutrient depletion, hypoxia, acidity, immune exclusion, and stromal remodeling are spatially patterned within tumors rather than evenly distributed across the tissue [55]. By combining molecular measurement with tissue architecture, spatial metabolomics can identify regional metabolic differences within the TME and help explain why neighboring tumor regions may differ in proliferation, immune infiltration, or treatment sensitivity [20]. Spatial multi-omics further strengthens this approach by integrating metabolite, lipid, and transcriptomic information within the same tissue context. By linking metabolites, lipids, and gene-expression programs to defined cellular neighborhoods, spatial multi-omics can uncover metabolic interactions within the TME and identify candidate vulnerabilities for precision therapy. For example, Sun et al. used spatial multi-omics approaches combining spatial transcriptomics with metabolomics and lipidomics to map cell-specific metabolic and transcriptional heterogeneity in gastric cancer; their findings suggested that metabolic remodeling is region- and cell-type-specific, with distinct molecular profiles at the tumor–normal interface and within immune-enriched regions [56]. Spatial multi-omics have similarly been used to predict response to immunotherapy in non-small cell lung cancer [57] and to characterize oligo-recurrent lung metastases in breast cancer patients [58]. However, as with single-cell approaches, candidate targets identified through spatial profiling should be interpreted as hypothesis-generating unless supported by functional validation in organoids, co-culture systems, xenografts, or genetically engineered models [59].

Multi-omics technologies provide a valuable framework for understanding cancer cell metabolic reprogramming as a spatially organized and cell-type-specific process. Single-cell methods define cellular heterogeneity, proteomic approaches reveal functional cell states, metabolomics and lipidomics capture biochemical pathway activity, and spatial technologies place these signals back into their tissue context. Ultimately, these advanced spatial and single-cell multi-omics platforms may eventually serve as the necessary analytical bridge to enable biomarker discovery, identify metabolic vulnerabilities and evaluate the efficacy of dietary and pharmacological interventions on tumor growth and patient survival outcomes. However, while preclinical tissue data is rich, human data currently rely predominantly on liquid biopsies rather than paired spatial tissue analysis, which, at present, limits the translational significance of multi-omics approaches.

## 5. Dietary Targeting of Metabolic Reprogramming

Dietary strategies provide a direct way to target cancer cell metabolic reprogramming by altering the nutrient environment in which tumor cells grow. Malignant cells rely on continuous nutrient availability to sustain proliferation, survival, and stress adaptation [60]. Interventions such as caloric restriction (CR), fasting, fasting-mimicking diets (FMDs), and nutrient-specific modulation may place pressure on metabolic pathways that support tumor progression. The biological rationale for these approaches is that modifying nutrient availability can interfere with nutrient-sensing pathways, growth-factor signaling, autophagy, and metabolic stress responses that cancer cells use to maintain growth under changing environmental conditions [22,28].

CR and fasting are among the most widely studied dietary approaches for manipulating systemic metabolism. Although both reduce nutrient availability, their biological effects are not identical, as tumor cells are exposed not only to changes in nutrient quantity, but also to changes in the timing, duration, and cyclic nature of nutrient stress [61]. Experimental work in mice has shown that fasting itself can drive many of the metabolic and molecular effects associated with CR, indicating that the timing and duration of nutrient deprivation are important determinants of systemic metabolic reprogramming [62].

FMDs provide important preclinical evidence that dietary intervention can expose cancer-specific metabolic vulnerabilities. In triple-negative breast cancer models, Salvadori et al. showed that FMD-induced stress activates starvation-escape pathways in tumor cells, including PI3K–AKT, mTOR, and CDK4/6, revealing compensatory signaling programs that can be identified and targeted pharmacologically [22]. Similar results were observed in models of hormone receptor-positive breast cancer, where FMDs enhanced the anti-tumor efficacy and reversed resistance to endocrine therapies with or without CDK4/6 inhibition [63]. Dietary restriction may also influence tumor development through host-mediated mechanisms, microbiome-derived metabolites, and immune remodeling, rather than direct nutrient deprivation alone. In a preclinical colorectal cancer model, Mao et al. showed that CR, but not intermittent fasting, protected against tumor formation through a mechanism linked to acetate production and accumulation of IFNγ+ CD8+ T cells in the tumor microenvironment, mediated by changes in the gut microbiota [28]. In addition to lowering blood glucose and insulin levels, CR lowers lipid availability in the tumor microenvironment and inhibits stearoyl-CoA desaturase (SCD) activity, which is required for the synthesis of monounsaturated fatty acids; this combination creates a metabolic vulnerability that slows tumor growth, in contrast to low-carbohydrate high-fat ketogenic diets that do not alter SCD activity and lipid availability in the TME [29].

Nutrient modulation offers an additional strategy for targeting cancer metabolic reprogramming. The metabolic state of cancer cells can predispose them to favor specific nutrients during transformation, making them vulnerable to changes in particular metabolites [64]. Adjusting dietary content according to these metabolic preferences may therefore restrict nutrient availability to cancer cells and limit disease progression [65]. However, this approach is likely to depend strongly on tumor type and tissue context, because local nutrient availability differs across organs and may determine whether systemic dietary changes meaningfully alter the metabolic environment surrounding the tumor [66].

The non-essential amino acids serine and glycine are heavily utilized by tumors for nucleotide synthesis, methylation, and antioxidant defense [67]. Dietary deprivation of serine and glycine forces cancer cells to upregulate de novo serine synthesis, a process that diverts glycolytic intermediates away from energy production; this metabolic rewiring drastically diminishes the cell’s antioxidant capacity, leading to the lethal accumulation of reactive oxygen species (ROS) [68]. Consequently, serine/glycine starvation exhibits profound synergy with chemotherapeutics that induce ROS, or in tumors lacking the antioxidant regulatory protein TP53-induced glycolysis and apoptosis regulator (TIGAR) [69]. Similarly, methionine is a critical precursor for S-adenosylmethionine, the universal methyl donor [70]. Gao et al. demonstrated that dietary methionine restriction profoundly alters human and murine metabolism, synergizing with targeted therapies and radiation by impairing nucleotide synthesis and altering tumor methylation patterns [71]. Methionine restriction may be more specific to cancer cells than serine/glycine restriction, which may widen the therapeutic index for clinical applications [72]. However, the systemic effects of methionine restriction are highly complex, as restricting methionine alters the composition of the gut microbiota, which can paradoxically suppress systemic antitumor immunity [73].

These considerations highlight important limitations of dietary modification therapies. Some tumors may be less responsive to dietary restriction if they can obtain nutrients from alternative sources, activate compensatory metabolic pathways, or survive through stress-response mechanisms such as autophagy [22,64]. In addition, cancer metabolism can resemble metabolic programs used by normal proliferating tissues, including immune cells involved in antitumor responses [40]. Therefore, dietary interventions designed to manipulate systemic nutrient availability may also affect normal tissues, immune function, and patient nutritional status [74]. This is particularly important when considering restrictive strategies in patients with advanced disease, cachexia risk, or reduced metabolic reserve.

**Figure 1:**
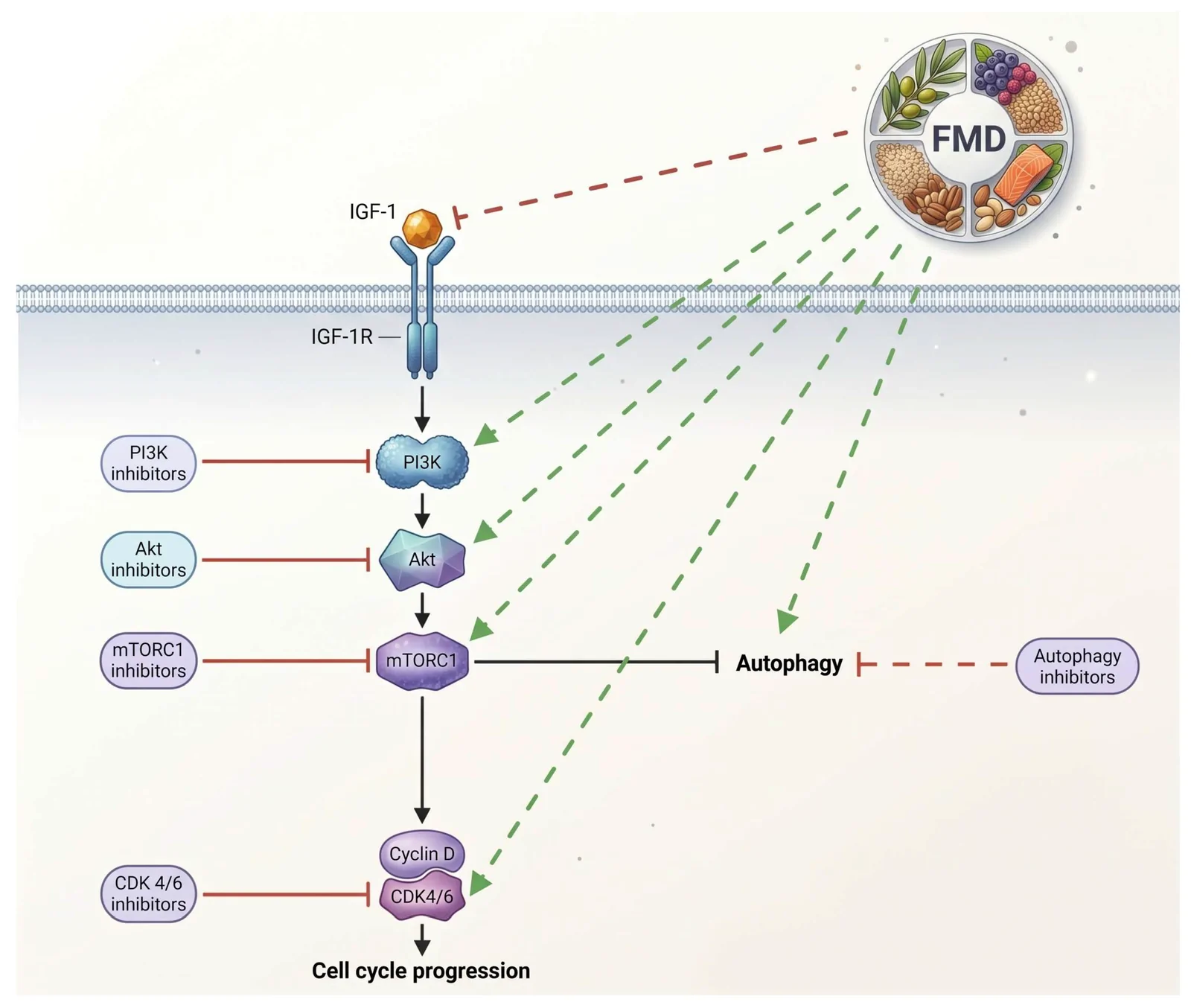
Dynamic interactions between fasting-mimicking diets, metabolic stress, and compensatory signaling networks in tumor cells. Green arrows indicate compensatory activation (starvation-escape pathways), while red flat-headed lines indicate pharmacological inhibition. FMD imposes systemic metabolic stress, leading to the paradoxical hyperactivation of PI3K, AKT, mTORC1, and CDK4/6 as a survival mechanism in differentiated tumor cells. This compensatory upregulation creates a therapeutic vulnerability that can be exploited by corresponding targeted kinase inhibitors. Approved inhibitors are denoted by solid borders, while investigational agents are denoted by dashed borders.

Overall, current evidence suggests that dietary strategies can influence cancer cell metabolic reprogramming through several interconnected mechanisms: reduction in nutrient and growth-factor availability, modulation of nutrient-sensing pathways, induction of metabolic stress, exposure of compensatory survival pathways, and remodeling of host immune or microbiome-dependent responses (Figure 1). However, the effectiveness of these strategies is unlikely to be universal. Future studies should prioritize primary in vivo, in vitro, and clinical evidence that directly links dietary intervention to changes in tumor metabolic pathways, treatment response, tumor microenvironment remodeling, and patient-specific safety outcomes.

## 6. Pharmacological Targeting of Metabolic Reprogramming

Pharmacological approaches to targeting cancer metabolic reprogramming use therapeutic agents to alter the pathways that control nutrient sensing, energy production, growth signaling, and cellular stress responses [75]. In this context, pharmacological mimetics are agents that reproduce or strengthen selected effects of dietary constraint; these effects may include reduced growth-factor signaling, inhibition of anabolic metabolism, or increased metabolic stress [76]. While dietary interventions can impose metabolic pressure on cancer cells, pharmacological mimetics offer a more controlled approach to modulating nutrient-sensing and stress-response pathways. Pharmacological agents enable more precise control of dosing, timing, and can be readily integrated with existing anticancer therapies to target defined metabolic vulnerabilities [77,78].

This approach is based on the principle that drugs can reproduce, intensify, or stabilize some of the metabolic effects induced by nutrient restriction, while also directly inhibiting defined molecular targets. Pathways such as insulin-like growth factor 1 receptor (IGF-1R)/insulin receptor (INSR) signaling, PI3K–AKT–mTOR activity, CDK4/6 signaling, and autophagy are particularly relevant because they regulate how cancer cells respond to nutrient availability, growth-factor withdrawal, and metabolic stress [79]. In addition to depriving tumor cells of nutrients, dietary restriction can expose adaptive dependencies that become pharmacologically actionable [80]. Recent preclinical evidence directly supports this link between dietary metabolic stress and pharmacological targeting. In triple-negative breast cancer models, FMD reduced glucose-dependent protein kinase A signaling and downregulated stemness-associated markers in cancer stem-like cells [22]. However, in differentiated triple-negative breast cancer (TNBC) cells, FMD activated starvation-escape pathways, including PI3K–AKT, mTOR, and CDK4/6 signaling [22]. RNA-seq analysis of SUM159 tumor masses further identified these compensatory pathways as druggable survival programs, and combining FMD with PI3K, AKT, and mTOR inhibitors enhanced cancer cell death and improved antitumor activity in mouse xenografts [22].

This concept has been further supported by recent work combining FMD with targeted therapies. Li et al. investigated whether FMD could overcome resistance to CDK4/6 inhibition in breast cancer [23]. In abemaciclib-resistant hormone receptor-positive cell lines, FMD-mimicking short-term starvation restored sensitivity to abemaciclib, while in TNBC models, FMD enhanced the growth-inhibitory effect of abemaciclib; these results were also validated in vivo in xenograft models [23]. Mechanistically, the combination suppressed NRAS- and IGF1-mediated mTORC1 signaling; mTORC1 activation is an established mechanism associated with acquired resistance to CDK4/6 inhibitors [81,82]. In this context, FMD acts not as a standalone therapy but as a metabolic sensitizer that may improve the effectiveness of pharmacological pathway inhibition [83].

mTOR inhibition also provides a pharmacological route for targeting nutrient-sensing metabolism. mTOR functions as a central regulator of anabolic growth and is responsive to nutrient and growth-factor availability [84]. Currently, mTOR inhibitors are used for the treatment of patients with selected breast, renal or neuroendocrine tumors [85,86]; nutritional status has been proposed as a biomarker for predicting response to mTOR inhibition [87,88]. The clinical utility of mTOR inhibitors has been hindered by early development of drug resistance; dietary metabolic interventions may have a role in this setting, particularly in tumors where activation of the PI3K–AKT–mTOR pathway may contribute to proliferation and treatment resistance, although clinical evidence is currently limited [89,90]. Furthermore, metabolic adverse events, such as hyperglycemia and hypercholesterolemia, are common during treatment with mTOR inhibitors [91]; dietary interventions may provide additional benefit by decreasing the frequency and/or severity of these events.

Insulin-like growth-factor (IGF) signaling represents another key bridge between nutrition and drug therapy. Because insulin and IGF-related pathways link systemic nutrient status to cancer cell proliferation and survival, pharmacological inhibition of IGF-1R/INSR signaling may mimic aspects of reduced nutrient and growth-factor availability [92]. Hassan et al. examined this principle by using BMS-754807, a dual IGF-1R/IR inhibitor, in an experimental model of esophageal adenocarcinoma as monotherapy and in combination with nab-paclitaxel [24]. BMS-754807 dose-dependently reduced tumor cell proliferation and increased apoptotic markers in vitro, including cleaved poly (ADP-ribose) polymerase (PARP) and cleaved caspase-3; in vivo, BMS-754807 reduced tumor growth and improved survival, while the combination with nab-paclitaxel further increased apoptosis and enhanced antitumor efficacy [24]. Although this study was not performed in a dietary intervention model, it supports the broader principle that blocking insulin/IGF-related signaling can impair tumor-promoting metabolic and survival pathways [93]. IGF-related signaling has also been shown to mediate resistance to mTOR inhibition; the combination of IGFR/IR and mTOR inhibition may delay or prevent treatment resistance [94].

Autophagy can function as an adaptive survival mechanism during nutrient stress. When cancer cells experience nutrient deprivation or pathway inhibition, autophagy may allow them to recycle intracellular components and maintain metabolic function [95]. Therefore, combining nutrient restriction, mTOR inhibition, or growth-factor pathway inhibition with autophagy blockade may reduce tumor cells’ ability to survive metabolic stress [96]. Knigin et al. tested this concept in lung neuroendocrine tumor models by treating tumor cells with chloroquine alone or in combination with mTOR inhibitors [25]. Chloroquine alone reduced tumor cell viability and proliferation and increased cytotoxicity and apoptosis, while combination with mTOR inhibitors augmented these effects. Mechanistically, chloroquine inhibited autophagic flux, and the combination with mTOR inhibition provided further evidence that autophagy blockade can potentiate metabolic pathway inhibition [25]. Autophagy inhibition may strengthen metabolic combination therapy when cancer cells depend on stress-adaptation mechanisms for survival [97]. Interestingly, autophagy has been consistently shown to be upregulated during fasting or CR and may impede their potential therapeutic benefit; inhibition of autophagy may increase the efficacy of dietary interventions in this setting [98].

Epigenetic regulation via microRNAs (miRNAs) plays a pivotal role in modulating both intrinsic cancer cell autophagy and the broader metabolic symbiosis within the TME [99]. In the context of autophagy, specific miRNAs may act either as tumor suppressors or oncogenes by targeting autophagy-related genes [100]. For instance, miR-30a has been identified as a potent tumor suppressor that negatively regulates the production of Beclin-1, a critical initiator of autophagosome formation [101]. Similarly, miR-372 has been shown to downregulate Unc-51 like autophagy activating kinase 1 (ULK1), effectively inhibiting the survival of highly aggressive tumor types, such as pancreatic adenocarcinoma, by disrupting autophagic flux [100]. Conversely, oncogenic miRNAs, such as miR-21, frequently exhibit overexpression in aggressive malignancies like glioblastoma, where they correlate with poor overall survival and modulate the PI3K/AKT/mTOR signaling cascade to support unchecked proliferation and autophagy-mediated drug resistance [102]. Beyond intrinsic autophagic regulation, miRNAs orchestrate metabolic reprogramming across the stromal compartment. miR-425-5p directly targets and suppresses MCT4 expression in CAFs; the disruption of MCT4-driven lactate efflux starves the adjacent tumor cells of their preferred energetic substrate, thereby reducing tumor cell viability, invasion, and angiogenic support [31]. Consequently, therapeutic strategies aiming to modulate specific miRNA networks, or employing pharmacological agents like N-acetylcysteine (NAC) to reduce oxidative stress-induced MCT4 expression in CAFs, may present novel avenues for disrupting the metabolic architecture of the TME [21].

The therapeutic targeting of lactate transport has rapidly advanced from preclinical models to human trials. The investigational MCT1 inhibitor AZD3965 was evaluated in a Phase I dose-escalation trial in patients with advanced solid tumors and lymphomas [103]. Pharmacodynamic assessments demonstrated target engagement, causing intracellular lactate accumulation and forcing tumors toward oxidative mitochondrial metabolism, establishing a recommended Phase II dose with manageable, reversible ocular toxicities [103]. Concurrently, the modulation of stromal MCT4 remains a high-priority target. While direct MCT4 inhibitors are limited, NAC has been shown to effectively suppress oxidative stress-induced MCT4 expression in CAFs in vitro, disrupting the lactate shuttle that fuels adjacent epithelial cancer cells [21].

While direct pharmacological inhibition of nutrient-sensing pathways offers precision, it frequently triggers systemic physiological feedback loops that can paradoxically protect tumor cells. For example, the administration of PI3K inhibitors profoundly disrupts insulin signaling in host tissues like skeletal muscle and the liver [104]. This disruption, and the resulting systemic hyperinsulinemia, effectively reactivates the PI3K-mTOR cascade directly in the tumor cells via the insulin receptor, thereby blunting the antitumor efficacy of the drug [104]. Crucially, this self-defeating systemic feedback can be therapeutically suppressed; co-administration of sodium-glucose cotransporter 2 (SGLT2) inhibitors, or the implementation of a carbohydrate-depleted ketogenic diet, rapidly lowers blood glucose and circulating insulin and may restore the efficacy of the PI3K inhibitor [105].

To consolidate these mechanistic insights, Table 3 summarizes the primary experimental evidence linking specific dietary and pharmacological interventions to their affected metabolic pathways and resulting cellular outcomes.

## 7. Clinical Safety and Efficacy of Dietary Modification Therapies in Solid Tumors

Although dietary targeting of cancer metabolism remains an emerging field, clinical translation is no longer purely theoretical. Early-phase studies have evaluated fasting, FMDs, caloric restriction, ketogenic approaches, and metabolic drugs as adjuncts to conventional anticancer therapy. Among these strategies, the most developed clinical evidence currently comes from FMD studies, in which short cycles of controlled dietary restriction have been tested alongside chemotherapy or standard systemic treatment.

In a proof-of-concept study, Vernieri et al. reported that cyclic FMD was safe and feasible in 101 patients with cancer receiving standard antitumor therapies [106]. The intervention consisted of repeated five-day cycles of severe but controlled caloric, carbohydrate, and protein restriction, delivered every three to four weeks for up to eight cycles. Importantly, the study showed that FMD induced systemic metabolic changes consistent with nutrient-sensing pathway modulation, including reductions in circulating glucose, insulin, and growth-factor signaling, while reshaping immune profiles in ways consistent with enhanced antitumor activity [106]. Similar metabolic outcomes were observed in a study of 90 patients with solid or hematological malignancies, where significant reductions in IGF-1, fat mass and insulin production were observed, without significant safety concerns [107]. These preliminary findings are significant, as they demonstrate that dietary metabolic intervention can produce measurable systemic and immune effects in patients, rather than remaining only a preclinical concept. However, because this was an early-phase, heterogeneous study across multiple tumor types and treatment regimens, it should be interpreted as evidence of feasibility and biological activity rather than definitive antitumor efficacy.

In breast cancer, the DIRECT randomized phase II trial provided more disease-specific clinical evidence by testing FMD as an adjunct to neoadjuvant chemotherapy in 131 patients with human epidermal growth factor receptor 2 (HER2)-negative stage II/III breast cancer [108]. Patients were randomized to receive either FMD or their regular diet for three days before and during neoadjuvant chemotherapy. The study did not show a clear reduction in chemotherapy-related toxicity, although patients in the FMD arm did not receive dexamethasone before anthracycline-containing chemotherapy cycles. In the intention-to-treat analysis, radiological complete or partial response occurred more frequently in the FMD group, whereas the per-protocol analysis suggested a higher likelihood of a major pathological response, defined as 90–100% tumor-cell loss in the resected specimen. The trial also reported metabolic changes among adherent participants, including lower insulin, glucose, and IGF-1 levels, increased ketone bodies, and reduced chemotherapy-induced DNA damage in T lymphocytes [108]. While the outcomes of DIRECT were encouraging, this study also highlights one of the important limitations of implementing dietary modifications in the clinical setting, as approximately 80% of patients failed to adhere to the study diet for at least half of the chemotherapy cycles, with only 21.5% completing the FMD across all chemotherapy cycles [108]. In an exploratory study in healthy individuals, two FMD cycles were enough to induce an immunomodulatory effect [109]; approximately 50% of patients were able to complete two FMD cycles in DIRECT [108]. In BREAKFAST, a smaller study of 30 patients with stage II/III TNBC, the majority of patients (73.3%) completed 8 FMD cycles; the cohort achieved a pathological complete response (pCR) rate of 56.7%; early downmodulation of tumor glycolysis was associated with a higher pCR rate [110]. It must be noted, however, that some patients in this trial concurrently received metformin, and efficacy data were measured against historical controls. It is important to note that the subset of TNBC patients included in the above studies did not receive the current standard-of-care therapy established by the KEYNOTE-522 study [111]; the potential impact of the addition of carboplatin and pembrolizumab to the treatment regimen on the feasibility and efficacy of FMD in this patient population requires further evaluation.

Comparable body composition changes were observed in a study of 30 Chinese breast cancer patients and in another study of 35 Irish prostate cancer patients, although compliance was higher in these cohorts, with over 80% of patients completing the protocol-specified regimen of three FMD cycles [112,113]. A pilot study of 10 patients with metastatic non-small cell lung cancer (NSCLC), mostly consisting of patients receiving oral tyrosine kinase inhibitors, also demonstrated the feasibility of FMD in this population, with 90% of patients completing one FMD cycle [114].

Several clinical trials are ongoing or recently registered, including studies of FMDs in heterogeneous malignancies, colorectal cancer recovery, chemotherapy toxicity, immunotherapy-related adverse events, and ketogenic diet combined with immunotherapy [115,116,117,118,119,120,121,122]. Importantly, multiple ongoing clinical trials utilize an “add-on” design; in this framework, the metabolic or lifestyle intervention is administered in addition to the standard-of-care systemic therapy, rather than as a standalone or replacement treatment. This ethical design was successfully championed by the phase III CHALLENGE trial, which recently established that structured exercise, when added to standard adjuvant chemotherapy for colon cancer, significantly reduced disease recurrence and improved overall survival without depriving patients of curative-intent medical therapies [123]. Notable examples of ongoing trials using this design include FASTIMMUNE, a phase II trial of maintenance atezolizumab plus cyclic FMD in patients with extensive stage small cell lung cancer after 4 cycles of induction chemoimmunotherapy [124] and BREAKFAST-2, a phase II trial of neoadjuvant chemoimmunotherapy plus cyclic FMD in patients with locally advanced TNBC [125] (Table 4).

Despite this progress, clinical translation faces several major challenges. First, dietary interventions are difficult to standardize because adherence, baseline nutritional status, tumor type, treatment regimen, body composition, metabolic health, and patient preference vary substantially among individuals and across differences in ethnicity and socioeconomic status [126]. Second, interventions designed to impose metabolic stress on tumor cells may also stress normal tissues, particularly in patients with cachexia, sarcopenia, gastrointestinal toxicity, frailty, or advanced disease. Third, many current studies remain small, heterogeneous, and underpowered for hard oncological endpoints such as progression-free survival and overall survival. Finally, a significant proportion of the early-phase FMD clinical data originates from a limited number of specialized research centers; independent, multi-center validation in broader, more diverse patient populations is needed to verify the generalizability of these findings and mitigate institutional bias. As a result, dietary metabolic interventions should be developed through carefully monitored clinical protocols with nutritional assessment, safety monitoring, and biomarker-defined endpoints.

## 8. Personalized Metabolic Oncology: Integrating TME Profiling and Nutritional Phenotyping

Cancer metabolic reprogramming is not uniform across tumor types, tissue sites, immune states, or nutritional phenotypes. Personalized metabolic oncology aims to match metabolic interventions to the tumor’s biological features and the patient’s physiological condition. A dietary or pharmacological metabolic strategy that is effective in one patient may be ineffective or unsafe in another if the tumor lacks the relevant metabolic dependency or if the patient has limited nutritional reserve. Personalization, therefore, requires two linked forms of stratification: profiling the tumor microenvironment to identify metabolic vulnerabilities and phenotyping the patient to determine whether a given metabolic intervention is clinically appropriate.

Integrated TME profiling provides one route for identifying these localized vulnerabilities. Spatial and single-cell technologies can reveal how metabolic states are distributed across tumor, immune, and stromal compartments rather than treating the tumor as a single homogeneous tissue. Hu et al. developed a single-cell, spatially resolved metabolic framework, termed scSpaMet, that combined untargeted spatial metabolomics with targeted multiplexed protein imaging in a single tissue-analysis pipeline [127]. Using this approach, the authors profiled human lung cancer, tonsil, and endometrial tissues and showed that metabolic features could be linked to specific cell phenotypes and spatial tissue organization. In lung cancer tissue, this enabled the identification of metabolic variation between tumor and stromal regions, as well as local metabolite competition between neighboring cell types [127]. Although this study was primarily methodological, it provides important proof of concept that spatial metabolic profiling can define cell-type-specific metabolic states in native human tissues. Similarly, Oliveira et al. used high-definition spatial transcriptomics in colorectal cancer to map immune cell populations and spatial niches within the TME [128]. Their study applied Visium HD to formalin-fixed paraffin-embedded colorectal cancer samples and integrated the spatial data with a single-cell RNA-sequencing reference atlas and Xenium in situ gene expression validation. This allowed the authors to identify distinct macrophage subpopulations at the tumor boundary, and to localize their interactions with tumor and T cell populations. Importantly, the study showed that immune cell identity and function are spatially organized within the TME, supporting the need for spatially informed profiling when selecting metabolic or immunometabolic interventions [128]. Together, these approaches show how TME profiling can move metabolic oncology beyond bulk pathway analysis and toward spatially informed therapeutic stratification.

Tumor-centered profiling alone is insufficient for personalized metabolic oncology because dietary and pharmacological metabolic interventions also act on the host. Nutritional phenotyping is therefore equally important. Patient-specific factors such as body composition, skeletal muscle mass, adiposity, cachexia risk, metabolic health, frailty, and treatment tolerance may determine whether restrictive dietary strategies are appropriate. Novel approaches to nutritional phenotyping include the use of computed tomography (CT) imaging, which is routinely performed for tumor assessments, to objectively measure body composition parameters such as skeletal muscle and adipose tissue. Borys et al. demonstrated the clinical relevance of this modality using automated CT-based body composition analysis in a large, pan-cancer cohort of patients with solid tumors [129]. The study derived a volumetric sarcopenia index from routinely acquired baseline CT imaging, defined as the ratio of muscle volume to bone volume, and evaluated its prognostic value using survival models and machine-learning approaches; a higher sarcopenia index was associated with prolonged overall survival in both the internal abdomen and thorax cohorts and remained significant in an external validation cohort [129]. Similarly, Han et al. reported that body-composition parameters, including skeletal muscle area, cachexia, and sarcopenia, were associated with prognosis in digestive tract cancers [130]. Circulating biomarkers may also be significant. The MAPK signaling pathway, particularly the mitogen-activated protein kinase kinase kinase 11 (MAP3K11)/growth differentiation factor 15 (GDF-15) axis, has been recognized as a key regulator of cancer cachexia; activation of this pathway has been associated with increased circulating levels of GDF-15 in animal models and in humans [131]. Importantly, elevated serum GDF-15 is increasingly being used to identify patients with cancer cachexia in clinical trials [132]. In this context, nutritional phenotyping is not simply supportive care; it becomes part of treatment stratification. Patients with low muscle mass, cachexia, or reduced metabolic reserve may be less suitable for restrictive dietary interventions and may require modified protocols, nutritional support, or non-restrictive pharmacological metabolic strategies [133]; the European Society for Clinical Nutrition and Metabolism (ESPEN) guidelines on nutrition in cancer patients explicitly caution against prolonged fasting and severe caloric restriction [134].

A personalized framework should therefore combine tumor metabolic profiling, TME mapping, and nutritional phenotyping to guide metabolic decision-making. Metabolic changes may occur before conventional radiological or clinical disease progression; metabolic signatures may provide early warning of drug resistance and allow for prompt treatment escalation or modification, before clinical deterioration occurs. Tumors with evidence of strong nutrient-sensing activity, metabolic stress adaptation, immune exclusion, hypoxia, or stromal metabolic crosstalk may be more suitable for metabolic intervention. In this model, precision metabolic oncology is characterized by the selection of the right metabolic intervention for the right tumor and the right patient, guided by integrated biological and nutritional data.

## 9. Conclusions

Metabolic reprogramming is a defining hallmark of cancer that enables malignant cells to adapt to environmental stressors and sustain unregulated proliferation. This dynamic process acts as a primary architect of the tumor microenvironment, driving critical processes such as immune exclusion, hypoxia adaptation, and stromal crosstalk. Multi-omics profiling can facilitate deconstructing this spatial and cellular complexity and may expose actionable metabolic dependencies within the tumor architecture. The exploitation of these vulnerabilities, via targeted dietary interventions like caloric restriction and fasting-mimicking diets, or through pharmacological mimetics, can provide a therapeutic strategy to disrupt oncogenic signaling and sensitize refractory tumors to standard therapies. Ultimately, the clinical viability of metabolic modulation rests on a dual mandate: exploiting tumor-specific vulnerabilities through high-resolution spatial profiling, while utilizing precise nutritional phenotyping to preserve host metabolic integrity, thereby maximizing the therapeutic window of these emerging interventions.

## Figures and Tables

**Table 1:** In vivo models used in the primary studies discussed in this review.

Model Category	Characteristic	Intervention Tested	References
**Human tumor-cell xenograft in immunodeficient mice**	Includes orthotopic or subcutaneous transplantation of human tumor cells.	Fasting/FMD, IGF-1R/IR inhibitors, PI3K/mTOR inhibitors, CDK4/6 inhibitors	[21,22,23,24,25]
**Murine syngeneic/allograft in immunocompetent mice**	Includes orthotopic or subcutaneous transplantation of murine tumor cells.	Fasting/FMD, Caloric Restriction, GPR4 targeting	[22,26,27,28]
**Murine tumor transplantation in immunodeficient mice**	Used to separate immune-dependent from tumor-intrinsic effects.	Caloric Restriction, GPR4 manipulation	[26,27]
**Spontaneous/genetically** **engineered mouse model**	MMTV-PyMT.	FMD + CDK4/6 inhibitor (Abemaciclib)	[23]
**LSL-Kras** (**G12D**) **genetically engineered mouse model**	Spontaneous pancreatic/lung tumor model.	Dietary Lipid Depletion/Ketogenic Diet	[29]
**Microbiome-depleted or** **microbiome-modified tumor model**	Antibiotic depletion, microbiota transfer, *B. bifidum* or acetate rescue.	Caloric restriction with *B. bifidum*/acetate rescue	[28]
**Intravenous experimental** **metastasis model**	4T1-luc intravenous model.	Fasting/FMD + PI3K/AKT/mTOR inhibitors	[22]
**Intraperitoneal dissemination/survival model**	OE19 intraperitoneal xenograft.	IGF-1R/IR inhibitor (BMS-754807)	[24]
**Human tumor-cell xenograft in immunodeficient mice**	COR-L103 subcutaneous models.	AZD3965 (MCT1 inhibitor)	[30]

Note: Categories are non-mutually exclusive because individual studies could employ more than one in vivo model. Abbreviations: CDK4/6, cyclin-dependent kinases 4 and 6; FMD, fasting-mimicking diet; GPR4, G protein-coupled receptor 4; IGF-1R, insulin-like growth factor 1 receptor; IR, insulin receptor; LSL, Lox-Stop-Lox; MCT1, monocarboxylate transporter 1; MMTV-PyMT, mouse mammary tumor virus-polyoma middle tumor-antigen; mTOR, mechanistic target of rapamycin; PI3K, phosphoinositide 3-kinase.

**Table 2:** In vitro models used in the primary studies discussed in this review.

Cellular Model	Origin	Intervention Tested	Model Class	References
**MC38**	Murine colorectal carcinoma	Caloric restriction, *B. bifidum* rescue, GPR4 manipulation	Tumor-derived	[26,27,28]
**SUM159**	Human TNBC	FMD, PI3K/AKT/mTOR inhibitors	Tumor-derived	[22,23]
**4T1**	Murine TNBC	FMD, PI3K/AKT/mTOR inhibitors	Tumor-derived	[22,26]
**MDA-MB-231**	Human TNBC	FMD + CDK4/6i, miR-425-5p modulation, N-acetylcysteine	Tumor-derived	[23,31]
**SW480**	Human colorectal cancer	AZD3965, GPR4 manipulation	Tumor-derived	[21,27]
**HUVECs**	Human endothelial support model	miR-425-5p modulation, MCT1 targeting	Non-tumor	[21,31]
**BT-549**	Human TNBC	miR-425-5p modulation, N-acetylcysteine	Tumor-derived	[31]
**CT26**	Murine colorectal carcinoma	Serine/glycine/methionine restriction	Tumor-derived	[32]
**E0771**	Murine breast cancer	Caloric restriction	Tumor-derived	[26]
**Flo-1**	Human esophageal adenocarcinoma	IGF-1R/IR inhibitor (BMS-754807)	Tumor-derived	[24]
**HCT116**	Human colorectal cancer	GPR4 manipulation, LOXL2/JAK2 inhibition	Tumor-derived	[27]
**LoVo**	Human colorectal cancer	Serine/glycine/methionine restriction	Tumor-derived	[32]
**MCF-7**	Human ER-positive breast cancer	FMD + CDK4/6 inhibitor (Abemaciclib)	Tumor-derived	[23]
**MCF7-AR**	Abemaciclib-resistant MCF-7 derivative	FMD + CDK4/6 inhibitor (Abemaciclib)	Tumor-derived	[23]
**NCI-H727**	Human lung neuroendocrine tumor	mTOR inhibition combined with chloroquine	Tumor-derived	[25]
**OE19**	Human esophageal adenocarcinoma	IGF-1R/IR inhibitor (BMS-754807)	Tumor-derived	[24]
**SK-GT-2**	Human esophageal adenocarcinoma	IGF-1R/IR inhibitor (BMS-754807)	Tumor-derived	[24]
**T47D**	Human ER-positive breast cancer	FMD + CDK4/6 inhibitor (Abemaciclib)	Tumor-derived	[23]
**T47D-AR**	Abemaciclib-resistant T47D derivative	FMD + CDK4/6 inhibitor (Abemaciclib)	Tumor-derived	[23]
**Patient-derived breast CAFs**	Patient-derived breast stromal model	miR-425-5p modulation, N-acetylcysteine	Non-tumor	[31]
**Normal fibroblasts**	Fibroblast control model	miR-425-5p modulation, N-acetylcysteine	Non-tumor	[31]
**MS5 stromal cells**	Stromal support model	miR-425-5p modulation	Non-tumor	[31]
**HFF-1 fibroblasts**	Human fibroblast support model	Serine/glycine/methionine restriction	Non-tumor	[32]

Note: Tumor-derived and non-tumor support models are reported separately. Abbreviations: AKT, protein kinase B; AR, abemaciclib-resistant; CAF, cancer-associated fibroblast; CDK4/6, cyclin-dependent kinases 4 and 6; ER, estrogen receptor; FMD, fasting-mimicking diet; GPR4, G protein-coupled receptor 4; HUVECs, human umbilical vein endothelial cells; IGF-1R, insulin-like growth factor 1 receptor; IR, insulin receptor; JAK2, Janus kinase 2; LOXL2, lysyl oxidase-like 2; MCT1, monocarboxylate transporter 1; mTOR, mechanistic target of rapamycin; PI3K, phosphoinositide 3-kinase; TNBC, triple-negative breast cancer.

**Table 3:** Affected metabolic pathways and targeted molecules by dietary and pharmacological interventions.

References	Intervention	Target	Affected Pathway/Molecule	Level of Evidence	Model System	Outcome
[22]	Fasting/FMD + PI3K/AKT/mTOR inhibitors	Glucose, Insulin	PI3K-AKT-mTORC1	Preclinical in vitro & in vivo	SUM159, 4T1 cells; tumor xenografts	Reduced CSC stemness; exposure of starvation-escape pathways; restored drug sensitivity
[23]	FMD + CDK4/6 inhibitor (Abemaciclib)	Glucose, IGF-1	CDK4/6, NRAS, phospho-S6	Preclinical in vitro & in vivo	Breast cancer cell lines; MMTV-PyMT mice	Reversal of acquired CDK4/6 resistance; reduced tumor growth.
[24]	IGF-1R/IR inhibitor (BMS-754807)	IGF-1 Signaling	IGF-1R, Cleaved PARP/Caspase-3	Preclinical in vitro & in vivo	Esophageal adenocarcinoma cells; intraperitoneal xenograft	Increased apoptosis; enhanced efficacy of nab-paclitaxel.
[31]	miR-425-5p modulation	Lactate; glucose uptake; ROS	MCT4-mediated export; MCT1-mediated uptake; PKM2; LDHA; NF-kB; HIF-1α; EMT-related proteins	Preclinical in vitro	TNBC cells, HUVECs, patient-derived CAFs	Reduced tumor-cell viability and migration; reduced invasion and angiogenic support
[21]	N-acetylcysteine	Oxidative stress (ROS); MCT1	ROS/TGF-β/p38MAPK pathway; lactate efflux	Preclinical in vitro	K562 cells, CAFs (NAC); SCLC/lymphoma cells, human trial (AZD3965)	Modulation of myeloid differentiation; reduced stromal lactate export; intracellular lactate accumulation
[25]	mTOR inhibition combined with chloroquine	Autophagic flux	mTOR; LC3-II; p62; lysosomal degradation	Preclinical in vitro & in vivo	Lung neuroendocrine cells (NCI-H727); xenografts	Reduced viability and proliferation; increased cytotoxicity and apoptosis
[28]	Caloric restriction with microbiome depletion or *B. bifidum*/acetate rescue	Acetate	*B. bifidum*-dependent immune–metabolic axis; IFNγ-positive CD8-positive T cells	Preclinical in vivo	Microbiome-modified MC38 murine model	Suppressed MC38 tumor growth; enhanced antitumor immune activity
[27]	Acidic TME; GPR4 manipulation; LOXL2/JAK2 inhibition	Extracellular acidity/pH; collagen deposition	GPR4; JAK2/STAT3; LOXL2; TGF-β	Preclinical in vitro & in vivo	Colorectal cancer cells (SW480, HCT116); murine transplantation models	Collagen reorganization and restricted intratumoral CD8-positive T cell infiltration

Note: The table summarizes primary experimental evidence linking dietary or pharmacological metabolic interventions to measurable metabolic states, molecular targets, and tumor or TME outcomes. Abbreviations: AKT, protein kinase B; CAF, cancer-associated fibroblast; CDK4/6, cyclin-dependent kinases 4 and 6; CSC, cancer stem cell; EMT, epithelial-to-mesenchymal transition; FMD, fasting-mimicking diet; GPR4, G protein-coupled receptor 4; HIF-1α, hypoxia-inducible factor 1 alpha; HUVECs, human umbilical vein endothelial cells; IFNγ, interferon gamma; IGF-1, insulin-like growth factor 1; IGF-1R, insulin-like growth factor 1 receptor; IR, insulin receptor; JAK2, Janus kinase 2; LC3-II, microtubule-associated protein 1A/1B-light chain 3; LDHA, lactate dehydrogenase A; LOXL2, lysyl oxidase-like 2; MCT1, monocarboxylate transporter 1; MCT4, monocarboxylate transporter 4; mTORC1, mechanistic target of rapamycin complex 1; NAC, N-acetylcysteine; NF-κB, nuclear factor kappa B; NRAS, neuroblastoma RAS viral oncogene homolog; p38MAPK, p38 mitogen-activated protein kinase; p62, sequestosome 1; PARP, poly (ADP-ribose) polymerase; PI3K, phosphoinositide 3-kinase; PKM2, pyruvate kinase M2; ROS, reactive oxygen species; SCLC, small cell lung cancer; STAT3, signal transducer and activator of transcription 3; TGF-β, transforming growth factor beta; TME, tumor microenvironment; TNBC, triple-negative breast cancer.

**Table 4:** Notable clinical trials for dietary and pharmacological metabolic interventions in solid tumors.

Reference	Phase	N	Tumor Type	Intervention & Concurrent Therapy	Compliance/Key Findings
**NCT03340935** [106]	I/II	101	Heterogeneous solid tumors	Cyclic FMD + standard systemic therapies	High feasibility. Safe; reduced blood glucose/insulin; reshaped systemic immunity.
**DIRECT/NCT02126449** [108]	II	131	HER2-negative early breast cancer	Cyclic FMD + neoadjuvant chemotherapy	Poor compliance; enhanced pathological response in adherent patients.
**BREAKFAST/NCT04248998** [110]	II	30	Stage II/III TNBC	Cyclic FMD ± metformin + neoadjuvant chemo	56.7% pCR rate compared to historical controls; correlated with early glycolysis downmodulation.
**NCT03595540** [107]	I/II	90	Solid/Hematological malignancies	Cyclic FMD + active treatment	Safe and feasible; significant reductions in fat mass, IGF-1, and insulin.
[112]	I	30	Breast cancer	FMD + chemotherapy	Safe with few adverse effects; acceptable weight loss; effectively reduced IGF-1 and inflammation.
**NCT04292041** [113]	I	35	Prostate cancer	FMD (4 days/month)	High compliance (29/35 completed). Safe; resulted in decreased weight, abdominal circumference, and blood pressure.
**NCT03700437** [114]	I	10	Metastatic NSCLC	FMD + standard therapies	Evaluated the feasibility, safety, and compliance with FMD in advanced lung cancer.
**FASTIMMUNE/NCT04845018** [124]	II	80	Extensive-stage SCLC	FMD + maintenance atezolizumab	*Currently Recruiting.*
**BREAKFAST-2/NCT05206981** [125]	II	130	Stage II-III TNBC	FMD + neoadjuvant chemoimmunotherapy	*Currently Recruiting.*
**NCT01791595** [103]	I	40	Advanced solid tumors and lymphoma	AZD3965 (MCT1 inhibitor) monotherapy	MTD established at 10 mg BID. On-target lactate accumulation. Reversible dose-limiting ocular toxicities.

Abbreviations: BID, bis in die (twice a day); FMD, fasting-mimicking diet; HER2, human epidermal growth factor receptor 2; IGF-1, insulin-like growth factor 1; MCT1, monocarboxylate transporter 1; MTD, maximum tolerated dose; pCR, pathological complete response; SCLC, small cell lung cancer; TNBC, triple-negative breast cancer.

## Data Availability

No new data were created or analyzed in this study. Data sharing is not applicable to this article.

## Abbreviations

AKT, protein kinase B; AR, abemaciclib-resistant; BID, bis in die (twice a day); CAF, cancer-associated fibroblast; CDK4/6, cyclin-dependent kinases 4 and 6; CR, caloric restriction; CSC, cancer stem cell; CT, computed tomography; CyTOF, mass cytometry; ECM, extracellular matrix; EMT, epithelial-to-mesenchymal transition; ER, estrogen receptor; ESPEN, European Society for Clinical Nutrition and Metabolism; FMD, fasting-mimicking diet; GDF-15, growth differentiation factor 15; GLUT1, glucose transporter 1; GPR4, G protein-coupled receptor 4; HER2, human epidermal growth factor receptor 2; HIF-1α, hypoxia-inducible factor 1 alpha; HIF-2α, hypoxia-inducible factor 2 alpha; HUVECs, human umbilical vein endothelial cells; IFNγ, interferon gamma; IGF-1, insulin-like growth factor 1; IGF-1R, insulin-like growth factor 1 receptor; INSR, insulin receptor; IR, insulin receptor; JAK2, Janus kinase 2; LC-MS/MS, liquid chromatography with tandem mass spectrometry; LC3-II, microtubule-associated protein 1A/1B-light chain 3; LDHA, lactate dehydrogenase A; LOXL2, lysyl oxidase-like 2; LSL, Lox-Stop-Lox; MAP3K11, mitogen-activated protein kinase kinase kinase 11; MAPK, mitogen-activated protein kinase; MCT1, monocarboxylate transporter 1; MCT4, monocarboxylate transporter 4; MMTV-PyMT, mouse mammary tumor virus-polyoma middle tumor-antigen; MTD, maximum tolerated dose; mTOR, mechanistic target of rapamycin; mTORC1, mechanistic target of rapamycin complex 1; NAC, N-acetylcysteine; NF-κB, nuclear factor kappa B; NRAS, neuroblastoma RAS viral oncogene homolog; NSCLC, non-small cell lung cancer; p38MAPK, p38 mitogen-activated protein kinase; p62, sequestosome 1; PARP, poly (ADP-ribose) polymerase; pCR, pathological complete response; PD-L1, programmed death-ligand 1; PI3K, phosphoinositide 3-kinase; PKA, protein kinase A; PKM2, pyruvate kinase M2; ROS, reactive oxygen species; SCD, stearoyl-CoA desaturase; SCLC, small cell lung cancer; scRNA-seq, single-cell RNA sequencing; SGLT2, sodium-glucose cotransporter 2; STAT3, signal transducer and activator of transcription 3; TGF-β, transforming growth factor beta; TIGAR, TP53-induced glycolysis and apoptosis regulator; TIL, tumor-infiltrating lymphocyte; TLR4, Toll-like receptor 4; TME, tumor microenvironment; TNBC, triple-negative breast cancer; ULK1, Unc-51 like autophagy activating kinase 1; VHL, von Hippel–Lindau.

## References

[1] Wu Q., Gao Z.-J., Yu X., Wang P. (2022). Dietary Regulation in Health and Disease. Signal Transduct. Target. Ther..

[2] Pavlova N.N., Zhu J., Thompson C.B. (2022). The Hallmarks of Cancer Metabolism: Still Emerging. Cell Metab..

[3] Moschandrea C., Frezza C. (2026). Metabolic Permissiveness: How Tissue Context Shapes Cancer. Genes Dev..

[4] Tan R.J.Y., Liu Y., Chen W., Liang Y., Ling G.S. (2026). Targeting Immunometabolism in Cancer through Pharmacological and Lifestyle Interventions. PLoS Biol..

[5] Chiu C.-F., Guerrero J., Regalado R., Zhou J., Notarte K., Lu Y.-W., Encarnacion P., Carles C., Octavo E., Limbaroc D. (2024). Insights into Metabolic Reprogramming in Tumor Evolution and Therapy. Cancers.

[6] Hanahan D. (2022). Hallmarks of Cancer: New Dimensions. Cancer Discov..

[7] Hanahan D., Weinberg R.A. (2011). Hallmarks of Cancer: The next Generation. Cell.

[8] Nong S., Han X., Xiang Y., Qian Y., Wei Y., Zhang T., Tian K., Shen K., Yang J., Ma X. (2023). Metabolic Reprogramming in Cancer: Mechanisms and Therapeutics. MedComm.

[9] Arner E.N., Rathmell J.C. (2023). Metabolic Programming and Immune Suppression in the Tumor Microenvironment. Cancer Cell.

[10] Bose S., Allen A.E., Locasale J.W. (2020). The Molecular Link from Diet to Cancer Cell Metabolism. Mol. Cell.

[11] Faubert B., Solmonson A., DeBerardinis R.J. (2020). Metabolic Reprogramming and Cancer Progression. Science.

[12] Xu Y., Liu X.-Y., Zhang H., Chen L., Wang H., Shao Z.-M., Xiao Y., Jiang Y.-Z. (2026). Comprehensive Single-Cell Metabolic Profiling Identifies Targets to Sensitize Triple-Negative Breast Cancer to Chemo-Immunotherapy. Cell Rep. Med..

[13] Wang Y., Xia Y., Lu Z. (2018). Metabolic Features of Cancer Cells. Cancer Commun..

[14] Ohshima K., Morii E. (2021). Metabolic Reprogramming of Cancer Cells during Tumor Progression and Metastasis. Metabolites.

[15] Demicco M., Liu X.-Z., Leithner K., Fendt S.-M. (2024). Metabolic Heterogeneity in Cancer. Nat. Metab..

[16] You M., Xie Z., Zhang N., Zhang Y., Xiao D., Liu S., Zhuang W., Li L., Tao Y. (2023). Signaling Pathways in Cancer Metabolism: Mechanisms and Therapeutic Targets. Signal Transduct. Target. Ther..

[17] Morita M., Kudo K., Shima H., Tanuma N. (2021). Dietary Intervention as a Therapeutic for Cancer. Cancer Sci..

[18] Kanarek N., Petrova B., Sabatini D.M. (2020). Dietary Modifications for Enhanced Cancer Therapy. Nature.

[19] Torrence M.E., Manning B.D. (2018). Nutrient Sensing in Cancer. Annu. Rev. Cancer Biol..

[20] Feng T., Piao H.-L., Chen D. (2026). Multi-Modal Metabolomics Deciphers Pan-Cancer Metabolic Landscapes and Spatial-Niche-Specific Alternations. Metabolites.

[21] Wang X., Qu Y., Ji J., Liu H., Luo H., Li J., Han X. (2024). Colorectal Cancer Cells Establish Metabolic Reprogramming with Cancer-Associated Fibroblasts (CAFs) through Lactate Shuttle to Enhance Invasion, Migration, and Angiogenesis. Int. Immunopharmacol..

[22] Salvadori G., Zanardi F., Iannelli F., Lobefaro R., Vernieri C., Longo V.D. (2021). Fasting-Mimicking Diet Blocks Triple-Negative Breast Cancer and Cancer Stem Cell Escape. Cell Metab..

[23] Li N., Sun Y.-J., Huang L.-Y., Li R.-R., Zhang J.-S., Qiu A.-H., Wang J., Yang L. (2025). Fasting-Mimicking Diet Potentiates Anti-Tumor Effects of CDK4/6 Inhibitors against Breast Cancer by Suppressing NRAS- and IGF1-Mediated mTORC1 Signaling. Drug Resist. Updat..

[24] Hassan M.S., Johnson C., Ponna S., Scofield D., Awasthi N., Von Holzen U. (2024). Inhibition of Insulin-like Growth Factor 1 Receptor/Insulin Receptor Signaling by Small-Molecule Inhibitor BMS-754807 Leads to Improved Survival in Experimental Esophageal Adenocarcinoma. Cancers.

[25] Knigin A., Avniel-Polak S., Leibowitz G., Oleinikov K., Gross D.J., Grozinsky-Glasberg S. (2021). The Autophagy Inhibitor Chloroquine, Alone or in Combination with mTOR Inhibitors, Displays Anti-Tumor Effects in In Vitro and In Vivo Lung Carcinoid Models. Cancers.

[26] Bailey C.M., Liu Y., Liu M., Du X., Devenport M., Zheng P., Liu Y., Wang Y. (2022). Targeting HIF-1α Abrogates PD-L1–Mediated Immune Evasion in Tumor Microenvironment but Promotes Tolerance in Normal Tissues. J. Clin. Investig..

[27] Bai S., Chen M., Wang X., Lan N., Meng R., Yan Y., Liu W., Li W., Wu F., Zhang X. (2026). GPR4 Promotes Immune Exclusion in Colon Cancer through LOXL2-Mediated Extracellular Matrix Remodeling. Nat. Commun..

[28] Mao Y.-Q., Huang J.-T., Zhang S.-L., Kong C., Li Z.-M., Jing H., Chen H.-L., Kong C.-Y., Huang S.-H., Cai P.-R. (2023). The Antitumour Effects of Caloric Restriction Are Mediated by the Gut Microbiome. Nat. Metab..

[29] Lien E.C., Westermark A.M., Zhang Y., Yuan C., Li Z., Lau A.N., Sapp K.M., Wolpin B.M., Vander Heiden M.G. (2021). Low Glycaemic Diets Alter Lipid Metabolism to Influence Tumour Growth. Nature.

[30] Polański R., Hodgkinson C.L., Fusi A., Nonaka D., Priest L., Kelly P., Trapani F., Bishop P.W., White A., Critchlow S.E. (2014). Activity of the Monocarboxylate Transporter 1 Inhibitor AZD3965 in Small Cell Lung Cancer. Clin. Cancer Res..

[31] Affinito A., Quintavalle C., Chianese R.V., Roscigno G., Fiore D., D’Argenio V., Thomas G., Savarese A., Ingenito F., Cocca L. (2024). MCT4-Driven CAF-Mediated Metabolic Reprogramming in Breast Cancer Microenvironment Is a Vulnerability Targetable by miR-425-5p. Cell Death Discov..

[32] Sun J., Zhao Z., Lu J., An W., Zhang Y., Li W., Yang L. (2024). The Tumor Microenvironment Mediates the HIF-1α/PD-L1 Pathway to Promote Immune Escape in Colorectal Cancer. Int. J. Mol. Sci..

[33] Wang Q., Shao X., Zhang Y., Zhu M., Wang F.X.C., Mu J., Li J., Yao H., Chen K. (2023). Role of Tumor Microenvironment in Cancer Progression and Therapeutic Strategy. Cancer Med..

[34] Sung Y., Kim D.K., Kim J.S., Kim S.-J., Kim J.H., Han J.M. (2026). Metabolic Networks in the Tumor Microenvironment: Roles of Amino Acid and Lipid Metabolism Pathways in Cancer Progression and Therapy. Exp. Mol. Med..

[35] Gajewski T.F., Schreiber H., Fu Y.-X. (2013). Innate and Adaptive Immune Cells in the Tumor Microenvironment. Nat. Immunol..

[36] Bruni S., Mercogliano M.F., Mauro F.L., Cordo Russo R.I., Schillaci R. (2023). Cancer Immune Exclusion: Breaking the Barricade for a Successful Immunotherapy. Front. Oncol..

[37] Chang M., Feng M., Lei Y., Wang Y., Bai L., Wang Y. (2026). Integrated Single-Cell and Spatial Transcriptomic Analysis Reveals mCAF-SPP1^+^ Macrophage-T Cell Crosstalk Shaping Immunosuppressive Niches in Colorectal Cancer Liver Metastasis. J. Transl. Med..

[38] Chen Y., McAndrews K.M., Kalluri R. (2021). Clinical and Therapeutic Relevance of Cancer-Associated Fibroblasts. Nat. Rev. Clin. Oncol..

[39] Zhang H., Liu S., Fu S., Zhao Q., Wang Y., Yuan Y., Zhang C. (2025). Novel Insight into the Warburg Effect: Sweet Temptation. Crit. Rev. Oncol./Hematol..

[40] Leone R.D., Powell J.D. (2020). Metabolism of Immune Cells in Cancer. Nat. Rev. Cancer.

[41] Xiao C., Tian H., Zheng Y., Yang Z., Li S., Fan T., Xu J., Bai G., Liu J., Deng Z. (2022). Glycolysis in Tumor Microenvironment as a Target to Improve Cancer Immunotherapy. Front. Cell Dev. Biol..

[42] Chen Z., Han F., Du Y., Shi H., Zhou W. (2023). Hypoxic Microenvironment in Cancer: Molecular Mechanisms and Therapeutic Interventions. Signal Transduct. Target. Ther..

[43] Bae T., Hallis S.P., Kwak M.-K. (2024). Hypoxia, Oxidative Stress, and the Interplay of HIFs and NRF2 Signaling in Cancer. Exp. Mol. Med..

[44] Rodriguez M., Qin Q., Tambar U.K., Brugarolas J. (2026). Targeting HIF-2α in Renal Cell Carcinoma: Expanding upon Belzutifan. Cancer.

[45] Jonasch E., Iliopoulos O., Rathmell W.K., Narayan V., Maughan B.L., Oudard S., Else T., Maranchie J.K., Welsh S.J., Bundsbæk Bøndergaard Iversen A. (2022). LITESPARK-004 (MK-6482-004) Phase 2 Study of Belzutifan, an Oral Hypoxia-Inducible Factor 2α Inhibitor (HIF-2α), for von Hippel-Lindau (VHL) Disease: Update with More than Two Years of Follow-up Data. J. Clin. Oncol..

[46] Choueiri T.K., Powles T., Peltola K., de Velasco G., Burotto M., Suarez C., Ghatalia P., Iacovelli R., Lam E.T., Verzoni E. (2024). Belzutifan versus Everolimus for Advanced Renal-Cell Carcinoma. N. Engl. J. Med..

[47] Choueiri T.K., Motzer R.J., Karam J.A., Yip W., Suárez C., Ye D., He Z., Caglevic C., Ferguson T., Chang Y.-H. (2026). Adjuvant Pembrolizumab plus Belzutifan for Renal-Cell Carcinoma. N. Engl. J. Med..

[48] Tharp K.M., Kersten K., Maller O., Timblin G.A., Stashko C., Canale F.P., Menjivar R.E., Hayward M.-K., Berestjuk I., ten Hoeve J. (2024). Tumor-Associated Macrophages Restrict CD8+ T Cell Function through Collagen Deposition and Metabolic Reprogramming of the Breast Cancer Microenvironment. Nat. Cancer.

[49] Chen W.W., Pacold M.E., Sabatini D.M., Kanarek N. (2025). Technologies for Decoding Cancer Metabolism with Spatial Resolution. Cold Spring Harb. Perspect. Med..

[50] Bica C., Zanoaga O., Pop L., Ciocan C., Raduly L., Nuțu A., Berindan-Neagoe I., Bender A. (2025). Tumor Heterogeneity Assessment Using Single-Cell RNA Sequencing (scRNA-Seq): Applications in Lung Cancer for Diagnosis and Treatment. Front. Immunol..

[51] Wang L., Liu Y., Dai Y., Tang X., Yin T., Wang C., Wang T., Dong L., Shi M., Qin J. (2023). Single-Cell RNA-Seq Analysis Reveals BHLHE40-Driven pro-Tumour Neutrophils with Hyperactivated Glycolysis in Pancreatic Tumour Microenvironment. Gut.

[52] Zhang N., Liu Q., Wang Q., Liu X., Zhang S., Tian X., Li L., Wang S., Lv B., Jiang S. (2025). Single-cell Transcriptomic Analysis Revealed the Tumor-associated Microenvironment of Papillary Thyroid Carcinoma with Metastasis. Oncol. Lett..

[53] Feng C., Wang X., Tao Y., Xie Y., Lai Z., Li Z., Hu J., Tang S., Pan L., He L. (2021). Single-Cell Proteomic Analysis Dissects the Complexity of Tumor Microenvironment in Muscle Invasive Bladder Cancer. Cancers.

[54] Kyros E., Mylonakis A., Kastanaki P., Velliou R.-I., Panagakis A., Davakis S., Dimitriou N., Felekouras E., Papalampros A. (2026). Cytometry by Time-of-Flight for Profiling Therapeutic Response in Colorectal Cancer: Strengths, Limitations, and Translational Challenges. Anticancer Res..

[55] Min X., Zhao Y., Yu M., Zhang W., Jiang X., Guo K., Wang X., Huang J., Li T., Sun L. (2024). Spatially Resolved Metabolomics: From Metabolite Mapping to Function Visualising. Clin. Transl. Med..

[56] Sun C., Wang A., Zhou Y., Chen P., Wang X., Huang J., Gao J., Wang X., Shu L., Lu J. (2023). Spatially Resolved Multi-Omics Highlights Cell-Specific Metabolic Remodeling and Interactions in Gastric Cancer. Nat. Commun..

[57] Aung T.N., Monkman J., Warrell J., Vathiotis I., Bates K.M., Gavrielatou N., Trontzas I.P., Tan C.W., Fernandez A.I., Moutafi M. (2025). Spatial Signatures for Predicting Immunotherapy Outcomes Using Multi-Omics in Non-Small Cell Lung Cancer. Nat. Genet..

[58] Gao Y., Li B., Jin Y., Cheng J., Tian W., Ying L., Hong L., Xin S., Lin B., Liu C. (2025). Spatial Multi-Omics Profiling of Breast Cancer Oligo-Recurrent Lung Metastasis. Oncogene.

[59] Du L., Yang H. (2025). Spatial Omics in 3D Culture Model Systems: Decoding Cellular Positioning Mechanisms and Microenvironmental Dynamics. J. Transl. Med..

[60] Martínez-Reyes I., Chandel N.S. (2021). Cancer Metabolism: Looking Forward. Nat. Rev. Cancer.

[61] Ibrahim E.M., Al-Foheidi M.H., Al-Mansour M.M. (2021). Energy and Caloric Restriction, and Fasting and Cancer: A Narrative Review. Support. Care Cancer.

[62] Pak H.H., Haws S.A., Green C.L., Koller M., Lavarias M.T., Richardson N.E., Yang S.E., Dumas S.N., Sonsalla M., Bray L. (2021). Fasting Drives the Metabolic, Molecular and Geroprotective Effects of a Calorie-Restricted Diet in Mice. Nat. Metab..

[63] Caffa I., Spagnolo V., Vernieri C., Valdemarin F., Becherini P., Wei M., Brandhorst S., Zucal C., Driehuis E., Ferrando L. (2020). Fasting-Mimicking Diet and Hormone Therapy Induce Breast Cancer Regression. Nature.

[64] Quek L.-E., van Geldermalsen M., Guan Y.F., Wahi K., Mayoh C., Balaban S., Pang A., Wang Q., Cowley M.J., Brown K.K. (2022). Glutamine Addiction Promotes Glucose Oxidation in Triple-Negative Breast Cancer. Oncogene.

[65] Kubota Y., Han Q., Aoki Y., Masaki N., Obara K., Hamada K., Hozumi C., Wong A.C.W., Bouvet M., Tsunoda T. (2023). Synergy of Combining Methionine Restriction and Chemotherapy: The Disruptive Next Generation of Cancer Treatment. Cancer Diagn. Progn..

[66] Abbott K.L., Subudhi S., Ferreira R., Gültekin Y., Steinbuch S.C., Munim M.B., Ramesh D.L., Honeder S.E., Kumar A.S., Wu M. (2026). Nutrient Requirements of Organ-Specific Metastasis in Breast Cancer. Nature.

[67] Sun W., Zhao E., Cui H. (2023). Target Enzymes in Serine-Glycine-One-Carbon Metabolic Pathway for Cancer Therapy. Int. J. Cancer.

[68] Maddocks O.D.K., Athineos D., Cheung E.C., Lee P., Zhang T., van den Broek N.J.F., Mackay G.M., Labuschagne C.F., Gay D., Kruiswijk F. (2017). Modulating the Therapeutic Response of Tumours to Dietary Serine and Glycine Starvation. Nature.

[69] Sánchez-Castillo A., Kampen K.R. (2024). Understanding Serine and Glycine Metabolism in Cancer: A Path towards Precision Medicine to Improve Patient’s Outcomes. Discov. Oncol..

[70] Sanderson S.M., Gao X., Dai Z., Locasale J.W. (2019). Methionine Metabolism in Health and Cancer: A Nexus of Diet and Precision Medicine. Nat. Rev. Cancer.

[71] Gao X., Sanderson S.M., Dai Z., Reid M.A., Cooper D.E., Lu M., Richie J.P., Ciccarella A., Calcagnotto A., Mikhael P.G. (2019). Dietary Methionine Influences Therapy in Mouse Cancer Models and Alters Human Metabolism. Nature.

[72] Miyashi Y., Ishiguro T., Han Q., Li S., Kang B.M., Bouvet M., Tome Y., Nishida K., Hoffman R.M. (2026). Direct Comparison of Serine Restriction on the Viability of Normal and Cancer Cells Demonstrates That Methionine Restriction Is More Cancer Specific. Front. Oncol..

[73] Ji M., Xu X., Xu Q., Hsiao Y.-C., Martin C., Ukraintseva S., Popov V., Arbeev K.G., Randall T.A., Wu X. (2023). Methionine Restriction-Induced Sulfur Deficiency Impairs Antitumour Immunity Partially through Gut Microbiota. Nat. Metab..

[74] Vernieri C., Ligorio F., Tripathy D., Longo V.D. (2024). Cyclic Fasting-Mimicking Diet in Cancer Treatment: Preclinical and Clinical Evidence. Cell Metab..

[75] Pyun W.Y., Park J.H., Roh J.W., Jeon D., Kim J., Paik J.E., Cho S.C., Park S.Y., Lim H., Kim H. (2026). Nutrient Availability Dictates Cancer Metabolism–Based Therapeutic Responses to Nononcology Drugs. Cancer Res..

[76] De-Leon-Covarrubias U.E., Perez-Trujillo J.J., Villa-Cedillo S.A., Martinez-Perez A.G., Montes-de-Oca-Saucedo C.R., Loera-Arias M.d.J., Garcia-Garcia A., Saucedo-Cardenas O., Montes-de-Oca-Luna R. (2024). Unlocking the Potential: Caloric Restriction, Caloric Restriction Mimetics, and Their Impact on Cancer Prevention and Treatment. Metabolites.

[77] Chaudhary S., Chaudhary M.R., Jena M.K., Rath P.K., Mishra B.P., Paital B., Vyas A., Sahoo D.K., Singh P., Mehdi M.M. (2025). Calorie Restriction Mimetics against Aging and Inflammation. Biogerontology.

[78] Almendáriz-Palacios C., Mousseau D.D., Eskiw C.H., Gillespie Z.E. (2020). Still Living Better through Chemistry: An Update on Caloric Restriction and Caloric Restriction Mimetics as Tools to Promote Health and Lifespan. Int. J. Mol. Sci..

[79] Tang X., Li G., Shi L., Su F., Qian M., Liu Z., Meng Y., Sun S., Li J., Liu B. (2021). Combined Intermittent Fasting and ERK Inhibition Enhance the Anti-Tumor Effects of Chemotherapy via the GSK3β-SIRT7 Axis. Nat. Commun..

[80] Pallavi R., Gatti E., Durfort T., Stendardo M., Ravasio R., Leonardi T., Falvo P., Duso B.A., Punzi S., Xieraili A. (2024). Caloric Restriction Leads to Druggable LSD1-Dependent Cancer Stem Cells Expansion. Nat. Commun..

[81] Yoshida A., Bu Y., Qie S., Wrangle J., Camp E.R., Hazard E.S., Hardiman G., de Leeuw R., Knudsen K.E., Diehl J.A. (2019). SLC36A1-mTORC1 Signaling Drives Acquired Resistance to CDK4/6 Inhibitors. Sci. Adv..

[82] Rodriguez M.J., Perrone M.C., Riggio M., Palafox M., Salinas V., Elia A., Salgueiro N.D., Werbach A.E., Marks M.P., Kauffman M.A. (2023). Targeting mTOR to Overcome Resistance to Hormone and CDK4/6 Inhibitors in ER-Positive Breast Cancer Models. Sci. Rep..

[83] Kan Z., Wen J., Bonato V., Webster J., Yang W., Ivanov V., Kim K.H., Roh W., Liu C., Mu X.J. (2025). Real-World Clinical Multi-Omics Analyses Reveal Bifurcation of ER-Independent and ER-Dependent Drug Resistance to CDK4/6 Inhibitors. Nat. Commun..

[84] Zhang H., Xiao X., Pan Z., Dokudovskaya S. (2025). mTOR Signaling Networks: Mechanistic Insights and Translational Frontiers in Disease Therapeutics. Signal Transduct. Target. Ther..

[85] Afinitor|European Medicines Agency (EMA). https://www.ema.europa.eu/en/medicines/human/EPAR/afinitor.

[86] Torisel|European Medicines Agency (EMA). https://www.ema.europa.eu/en/medicines/human/EPAR/torisel.

[87] Pizzuti L., Marchetti P., Natoli C., Gamucci T., Santini D., Scinto A.F., Iezzi L., Mentuccia L., D’Onofrio L., Botticelli A. (2017). Fasting Glucose and Body Mass Index as Predictors of Activity in Breast Cancer Patients Treated with Everolimus-Exemestane: The EverExt Study. Sci. Rep..

[88] Ranallo N., Iamurri A.P., Foca F., Liverani C., De Vita A., Mercatali L., Calabrese C., Spadazzi C., Fabbri C., Cavaliere D. (2022). Prognostic and Predictive Role of Body Composition in Metastatic Neuroendocrine Tumor Patients Treated with Everolimus: A Real-World Data Analysis. Cancers.

[89] Koundouros N., Blenis J. (2022). Targeting mTOR in the Context of Diet and Whole-Body Metabolism. Endocrinology.

[90] Browne I.M., Okines A.F.C. (2024). Resistance to Targeted Inhibitors of the PI3K/AKT/mTOR Pathway in Advanced Oestrogen-Receptor-Positive Breast Cancer. Cancers.

[91] Arena C., Bizzoca M.E., Caponio V.C.A., Troiano G., Zhurakivska K., Leuci S., Lo Muzio L. (2021). Everolimus Therapy and Side-effects: A Systematic Review and Meta-analysis. Int. J. Oncol..

[92] Simpson A., Petnga W., Macaulay V.M., Weyer-Czernilofsky U., Bogenrieder T. (2017). Insulin-Like Growth Factor (IGF) Pathway Targeting in Cancer: Role of the IGF Axis and Opportunities for Future Combination Studies. Target. Oncol..

[93] Obr A.E., Kumar S., Chang Y.-J., Bulatowicz J.J., Barnes B.J., Birge R.B., Lazzarino D.A., Gallagher E., LeRoith D., Wood T.L. (2018). Insulin-like Growth Factor Receptor Signaling in Breast Tumor Epithelium Protects Cells from Endoplasmic Reticulum Stress and Regulates the Tumor Microenvironment. Breast Cancer Res..

[94] Yoon S.-O., Shin S., Karreth F.A., Buel G.R., Jedrychowski M.P., Plas D.R., Dedhar S., Gygi S.P., Roux P.P., Dephoure N. (2017). Focal Adhesion- and IGF1R-Dependent Survival and Migratory Pathways Mediate Tumor Resistance to mTORC1/2 Inhibition. Mol. Cell.

[95] Niu X., You Q., Hou K., Tian Y., Wei P., Zhu Y., Gao B., Ashrafizadeh M., Aref A.R., Kalbasi A. (2025). Autophagy in Cancer Development, Immune Evasion, and Drug Resistance. Drug Resist. Updat..

[96] Lu Y., Li A., Liu A., Li M., Wang M. (2026). Autophagy in Cancer: Context-Dependent Regulation and Precision Nanomedicine-Enabled Therapeutic Targeting. Biomedicines.

[97] Chi K.-H., Wang Y.-S., Huang Y.-C., Chiang H.-C., Chi M.-S., Chi C.-H., Wang H.-E., Kao S.-J. (2016). Simultaneous Activation and Inhibition of Autophagy Sensitizes Cancer Cells to Chemotherapy. Oncotarget.

[98] Bagherniya M., Butler A.E., Barreto G.E., Sahebkar A. (2018). The Effect of Fasting or Calorie Restriction on Autophagy Induction: A Review of the Literature. Ageing Res. Rev..

[99] Aseervatham J. (2023). Dynamic Role of Exosome microRNAs in Cancer Cell Signaling and Their Emerging Role as Noninvasive Biomarkers. Biology.

[100] Webb J.D., Shepherd T.G. (2026). ULK1’s Role in Cancer Progression and Its Emerging Therapeutic Potential. Cancer Biol. Ther..

[101] Wei J., Wang X., Yu D., Tu Y., Yu Y. (2024). MicroRNA-Mediated Autophagy and Drug Resistance in Cancer: Mechanisms and Therapeutic Strategies. Discov. Oncol..

[102] Molavand M., Ebrahimnezhade N., Kiani A., Yousefi B., Nazari A., Majidinia M. (2024). Regulation of Autophagy by Non-Coding RNAs in Human Glioblastoma. Med. Oncol..

[103] Halford S., Veal G.J., Wedge S.R., Payne G.S., Bacon C.M., Sloan P., Dragoni I., Heinzmann K., Potter S., Salisbury B.M. (2023). A Phase I Dose-Escalation Study of AZD3965, an Oral Monocarboxylate Transporter 1 Inhibitor, in Patients with Advanced Cancer. Clin. Cancer Res..

[104] Hopkins B.D., Pauli C., Du X., Wang D.G., Li X., Wu D., Amadiume S.C., Goncalves M.D., Hodakoski C., Lundquist M.R. (2018). Suppression of Insulin Feedback Enhances the Efficacy of PI3K Inhibitors. Nature.

[105] Noch E.K., Palma L.N., Yim I., Bullen N., Qiu Y., Ravichandran H., Kim J., Rendeiro A., Davis M.B., Elemento O. (2023). Insulin Feedback Is a Targetable Resistance Mechanism of PI3K Inhibition in Glioblastoma. Neuro Oncol..

[106] Vernieri C., Fucà G., Ligorio F., Huber V., Vingiani A., Iannelli F., Raimondi A., Rinchai D., Frigè G., Belfiore A. (2022). Fasting-Mimicking Diet Is Safe and Reshapes Metabolism and Antitumor Immunity in Patients with Cancer. Cancer Discov..

[107] Valdemarin F., Caffa I., Persia A., Cremonini A.L., Ferrando L., Tagliafico L., Tagliafico A., Guijarro A., Carbone F., Ministrini S. (2021). Safety and Feasibility of Fasting-Mimicking Diet and Effects on Nutritional Status and Circulating Metabolic and Inflammatory Factors in Cancer Patients Undergoing Active Treatment. Cancers.

[108] de Groot S., Lugtenberg R.T., Cohen D., Welters M.J.P., Ehsan I., Vreeswijk M.P.G., Smit V.T.H.B.M., de Graaf H., Heijns J.B., Portielje J.E.A. (2020). Fasting Mimicking Diet as an Adjunct to Neoadjuvant Chemotherapy for Breast Cancer in the Multicentre Randomized Phase 2 DIRECT Trial. Nat. Commun..

[109] de Gruil N., Welters M.J.P., Santegoets S.J., Boekestijn S., Kaart S., Pijl H., Kroep J.R., van der Burg S.H. (2026). Exploring the Immunomodulatory Effects of Fasting-Mimicking Diet in Healthy Individuals: A Pilot Study. Nutrients.

[110] Ligorio F., Vingiani A., Torelli T., Sposetti C., Drufuca L., Iannelli F., Zanenga L., Depretto C., Folli S., Scaperrotta G. (2025). Early Downmodulation of Tumor Glycolysis Predicts Response to Fasting-Mimicking Diet in Triple-Negative Breast Cancer Patients. Cell Metab..

[111] Schmid P., Cortes J., Pusztai L., McArthur H., Kümmel S., Bergh J., Denkert C., Park Y.H., Hui R., Harbeck N. (2020). Pembrolizumab for Early Triple-Negative Breast Cancer. N. Engl. J. Med..

[112] Xue M., Zhang N., Dong X., Tian Q., Shen K., Wu B. (2025). The Feasibility and Safety of Fasting-Mimicking Diet in Breast Cancer Patients with Chemotherapy in China. Breast Cancer Res. Treat..

[113] Fay-Watt V., O’Connor S., Roshan D., Romeo A.C., Longo V.D., Sullivan F.J. (2023). The Impact of a Fasting Mimicking Diet on the Metabolic Health of a Prospective Cohort of Patients with Prostate Cancer: A Pilot Implementation Study. Prostate Cancer Prostatic Dis..

[114] Ghaddar F., Ahmed S., Kirsch B., Jennewein L., Uhrich M.M., Althouse S.K., Longo V., Jalal S. (2026). Brief Report: A Pilot Study Evaluating the Feasibility, Safety, and Compliance with a Fasting-Mimicking Diet in Patients with Metastatic Non-Small Cell Lung Cancer. Clin. Lung Cancer.

[115] Redfern A. The FASTEX Trial: Fasting Mimicking Diet and Exercise Medicine as Adjuvant Therapies in the Treatment of Patients with Breast Cancer. https://www.ancora.ai/en/details/ACTRN12623000996662.

[116] Braud d.F. Impact of Dietary Intervention on Tumor Immunity: The DigesT Trial. https://clinicaltrials.gov/study/NCT03454282.

[117] Jenkins-Vogel T. A Randomized Controlled Study of a Fasting Mimicking Diet (FMD) in Conjunction with Combination Carboplatin and Paclitaxel in the Treatment of Patients with Advanced or Recurrent Ovarian, Fallopian Tube and Primary Peritoneal Cancer. https://clinicaltrials.gov/study/NCT05921149?viewType=Card&titles=A%20Randomized%20Controlled%20Study%20of%20a%20Fasting%20Mimicking%20Diet%20(FMD)%20in%20Conjunction%20With%20Combination%20Carboplatin%20&rank=1.

[118] Braud d.F. Safety, Feasibility and Metabolic Effects of the Fasting Mimicking Diet (FMD) in Cancer Patients. https://clinicaltrials.gov/study/NCT03340935?viewType=Card&titles=Safety,%20Feasibility%20and%20Metabolic%20Effects%20of%20the%20Fasting%20Mimicking%20Diet%20(FMD)%20in%20Cancer%20Patients&rank=1.

[119] Miao C. Effect of Perioperative Dietary Restriction Strategy on Postoperative Recovery and Outcomes of Patients with Colorectal Cancer: A Multicenter Prospective, Randomized, Controlled Study. https://clinicaltrials.gov/study/NCT05384444?viewType=Card&titles=Effect%20of%20Perioperative%20Dietary%20Restriction%20Strategy%20on%20Postoperative%20Recovery%20and%20Outcomes%20of%20Patients%20With%20Colorectal%20Cancer&rank=1.

[120] Mayo Clinic Effect of Fasting Mimicking Diet (FMD) on Immune Related Adverse Events for Cancer Patients on Immune Checkpoint Inhibitors: The FMD-ICI Feasibility Pilot Study. https://clinicaltrials.gov/study/NCT06438588?viewType=Card&titles=Effect%20of%20Fasting%20Mimicking%20Diet%20(FMD)%20on%20Immune%20Related%20Adverse%20Events%20for%20Cancer%20&rank=1.

[121] Markel G. Investigating the Effect of Ketogenic Diet on Immunological Parameters in Advanced Cancer Patients Undergoing Immunotherapy. https://clinicaltrials.gov/study/NCT06896552?viewType=Card&titles=Investigating%20the%20Effect%20of%20Ketogenic%20Diet%20on%20Immunological%20Parameters%20in%20Advanced%20Cancer%20Patients%20&rank=1.

[122] L-Nutra Inc. Use of a Fasting Mimicking Diet in Patients Undergoing Chemotherapy for Gynecologic Malignancies. https://clinicaltrials.gov/study/NCT06376604?viewType=Card&titles=Use%20of%20a%20Fasting%20Mimicking%20Diet%20in%20Patients%20Undergoing%20Chemotherapy%20for%20Gynecologic%20Malignancies&rank=1.

[123] Courneya K.S., Vardy J.L., O’Callaghan C.J., Gill S., Friedenreich C.M., Wong R.K.S., Dhillon H.M., Coyle V., Chua N.S., Jonker D.J. (2025). Structured Exercise after Adjuvant Chemotherapy for Colon Cancer. N. Engl. J. Med..

[124] Fondazione IRCCS Istituto Nazionale dei Tumori, Milano FASTing-like Approach to Improve the Efficacy of Maintenance IMMunotherapy in Extensive-Stage Small Cell Lung Cancer Patients Not Progressing on Chemoimmunotherapy Induction: The FASTIMMUNE Trial. https://clinicaltrials.gov/study/NCT05703997?viewType=Card&titles=FASTing-like%20Approach%20to%20Improve%20the%20Efficacy%20of%20Maintenance%20IMMunotherapy%20in%20Extensive-&rank=1.

[125] Vernieri C., Ligorio F., Dieci M.V., Lambertini M., De Angelis C., Iorfida M., Botticelli A., Strina C., Vingiani A., Provenzano L. (2023). 352TiP Neoadjuvant Chemo-Immunotherapy plus/Minus Fasting-like Approach in Stage II-III Triple-Negative Breast Cancer Patients: The Phase II Randomized BREAKFAST-2 Trial. Ann. Oncol..

[126] Martínez-Garay C., Djouder N. (2023). Dietary Interventions and Precision Nutrition in Cancer Therapy. Trends Mol. Med..

[127] Hu T., Allam M., Cai S., Henderson W., Yueh B., Garipcan A., Ievlev A.V., Afkarian M., Beyaz S., Coskun A.F. (2023). Single-Cell Spatial Metabolomics with Cell-Type Specific Protein Profiling for Tissue Systems Biology. Nat. Commun..

[128] de Oliveira M.F., Romero J.P., Chung M., Williams S.R., Gottscho A.D., Gupta A., Pilipauskas S.E., Mohabbat S., Raman N., Sukovich D.J. (2025). High-Definition Spatial Transcriptomic Profiling of Immune Cell Populations in Colorectal Cancer. Nat. Genet..

[129] Borys K., Haubold J., Keyl J., Bali M.A., De Angelis R., Boni K.B., Coquelet N., Kohnke J., Baldini G., Kroll L. (2025). Leveraging Sarcopenia Index by Automated CT Body Composition Analysis for Pan Cancer Prognostic Stratification. npj Digit. Med..

[130] Han J., Liu X., Wang J., Tang M., Xu J., Tan S., Liu X., Wu G. (2024). Prognostic Value of Body Composition in Patients with Digestive Tract Cancers: A Prospective Cohort Study of 8,267 Adults from China. Clin. Nutr. ESPEN.

[131] Lerner L., Tao J., Liu Q., Nicoletti R., Feng B., Krieger B., Mazsa E., Siddiquee Z., Wang R., Huang L. (2016). MAP3K11/GDF15 Axis Is a Critical Driver of Cancer Cachexia. J. Cachexia Sarcopenia Muscle.

[132] Crawford J., Groarke J., Collins S., Lubaczewski S., Roeland E.J., Naito T., Hendifar A., Fallon M.T., Takayama K., Asmis T.R. (2025). LBA102 Efficacy and Safety of Ponsegromab in Patients with Cancer-Associated Cachexia: Results from the Open-Label Extension of a Randomized, Placebo-Controlled, Phase II Study. Ann. Oncol..

[133] Setiawan T., Sari I.N., Wijaya Y.T., Julianto N.M., Muhammad J.A., Lee H., Chae J.H., Kwon H.Y. (2023). Cancer Cachexia: Molecular Mechanisms and Treatment Strategies. J. Hematol. Oncol..

[134] Muscaritoli M., Arends J., Bachmann P., Baracos V., Barthelemy N., Bertz H., Bozzetti F., Hütterer E., Isenring E., Kaasa S. (2021). ESPEN Practical Guideline: Clinical Nutrition in Cancer. Clin. Nutr..

